# DeepEGFR a graph neural network for bioactivity classification of EGFR inhibitors

**DOI:** 10.1038/s41598-025-22126-8

**Published:** 2025-10-31

**Authors:** Aijaz Ahmad Malik, Costerwell Khyriem, Sven Hauns, Imran Khan, Frederico G. Pinto, Azzat Al-Sadi, Rasheed Mohammad, Van Dinh Tran, Rolf Backofen, Nelson Soares, Mohammed Uddin, Omer S. Alkhnbashi

**Affiliations:** 1 Center for Applied and Translational Genomics, Mohammed Bin Rashid University of Medicine and Health Sciences, P.O. Box 505055, Dubai, United Arab Emirates; 2 College of Medicine, Mohammed Bin Rashid University of Medicine and Health Sciences, P.O. Box 505055, Dubai, United Arab Emirates; 3 Bioinformatics Group, Department of Computer Science, University of Freiburg, 79085 Freiburg, Germany; 4 Perth Children’s Hospital, Telethon Kids Institute, Perth, Australia; 5 Department of Chemistry, Federal University of Viçosa, Minas Gerais, Brazil; 6 Department of Computer Engineering, Hadhramout University, Hadhramout, Yemen; 7 Department of Computer Sciences, College of Computing and Digital Technology, Birmingham City University, Birmingham, B4 7XG UK; 8 Information and Computer Science Department, King Fahd University of Petroleum and Minerals, Dhahran, 31261 Saudi Arabia; 9 Signalling Research Centres BIOSS and CIBSS, University of Freiburg, 79085 Freiburg, Germany

**Keywords:** EGFR inhibitors, Graph neural networks (GNN), Fingerprints, Molecular docking, Substructures, Targeted cancer therapy, Computational models, Data acquisition, Data integration, Data processing, Functional clustering, Machine learning, Protein analysis, Biologics

## Abstract

Epidermal Growth Factor Receptor (EGFR) plays a critical role in the development of several cancers. Thus, modulation/inhibition of EGFR activity is an appealing target of developing novel cancer therapeutics. With the advent of modern machine learning technologies, it is now possible to simulate interactions with high precision between EGFR and small molecules to predict inhibitory/ modulatory activity at an unprecedented scale. In this work, we propose a novel machine-learning method to fast and precise classification of small compounds that are active, intermediate or inactive in inhibiting/modulating EGFR activity. We developed DeepEGFR, a novel multi-class graph neural network (GNN) model, to classify compounds into Active, Inactive, and Intermediate functional categories. DeepEGFR leverages complementary molecular representations, combining SMILES strings and molecular fingerprint matrices (Klekota-Roth and PubChem) to capture both structural and property-based features of compounds. The model constructs an advanced molecular graph representing atom type, formal charge, bond type, and bond order, through nodes and edges. DeepEGFR achieved superior performance compared to baseline machine learning algorithms (e.g., SVM, Random Forest, ANN), with approximately 94% F1-scores across training and test datasets for all activity classes. To ensure interpretability, the top 20 features identified by DeepEGFR were validated against the five key characteristics of FDA-approved EGFR inhibitors (Afatinib, Gefitinib, Osimertinib, Dacomitinib, Erlotinib), confirming the biological relevance of the features. Moreover, DeepEGFR successfully identified 300 underexplored EGFR-targeting compounds, demonstrating its potential to accelerate the discovery of therapeutic agents. These results highlight the effectiveness of graph neural networks in advancing molecular activity classification, setting a potential new benchmark for EGFR inhibitor prediction. These findings demonstrate the DeepEGFR’s ability to highlight the promising EGFR inhibitors, that have received limited prior investigation, thereby supporting its role in facilitating the rational development of targeted therapies for precision oncology.

**Supplementary Information:**

The online version contains supplementary material available at 10.1038/s41598-025-22126-8.

## Introduction

Lung cancer, particularly the non-small cell lung cancer (NSCLC) subtype, remains a significant cause of cancer-related mortality globally^1^. A key driver in NSCLC development is the presence of mutations within the epidermal growth factor receptor (EGFR) gene, making it a prominent therapeutic target. EGFR, a transmembrane receptor tyrosine kinase, plays a crucial role in regulating cell growth, proliferation, and differentiation. Upon ligand binding, such as with epidermal growth factor (EGF), EGFR undergoes dimerization and autophosphorylation, subsequently activating downstream signaling pathways, notably the RAS/MAPK and PI3K/AKT pathways^2^. These pathways govern diverse cellular processes, including cell cycle progression, apoptosis, and angiogenesis. In NSCLC, EGFR mutations, primarily located in exons 19 and 21, result in constitutive, ligand-independent receptor activation^3^. This aberrant activation promotes uncontrolled cell growth and proliferation, thus contributing to tumor development and progression. Approximately 10–15% of NSCLC patients exhibit EGFR mutations, with a higher prevalence observed in specific populations, including East Asians and never-smokers^4^. While recent GNN-based cheminformatics studies have achieved impressive results in molecular property prediction and drug–target interaction, no previous work has combined SMILES-derived molecular graphs with interpretable fingerprint descriptors in a multi-class EGFR QSAR framework. Existing GNN pipelines either focus solely on graph embeddings or use fixed fingerprint inputs, limiting mechanistic interpretability and multi-class activity discrimination. Liu, Moroz, & Isayev, (2023) demonstrated improved reaction-yield prediction using SMILES-based GNNs pre-trained on large molecular corpora, and a 2024 bibliometric analysis highlights expanding GNN applications in drug–target and drug–drug interaction tasks. However, these efforts do not address the unique challenges of classifying EGFR inhibitors into active, intermediate, and inactive categories, nor do they integrate multiple molecular representations. EGFR-TKIs have transformed NSCLC treatment, yet resistance mutations (e.g., T790M, C797S) emerge in over 50% of patients on first- and second-generation inhibitors. By uncovering 300 underexplored compounds with high predicted potency; including against T790M and C797S variants- DeepEGFR aims to expand the therapeutic arsenal for precision oncology.

EGFR, a member of the ErbB family of receptor tyrosine kinases (RTKs), which also includes ErbB2 (HER2), ErbB3, and ErbB4, is essential for cell growth, survival, proliferation, and differentiation.Structurally, EGFR comprises several distinct functional domains that coordinate ligand binding, receptor activation, and signal transduction. The extracellular domain (ECD), responsible for ligand binding, consists of four subdomains (I-IV). Subdomains I and III directly interact with ligands like EGF and transforming growth factor-alpha (TGF-α), forming the ligand-binding site. Subdomain II contains a dimerization arm crucial for receptor-receptor interactions upon ligand binding, while subdomain IV regulates conformational changes that facilitate activation. In the absence of ligand, EGFR adopts a tethered conformation, concealing the dimerization arm and preventing spontaneous dimerization. Ligand binding induces a conformational change to an extended conformation, exposing the dimerization arm and promoting dimerization. The transmembrane domain, a single alpha-helix spanning the plasma membrane, stabilizes receptor dimerization and transmits conformational changes from the ECD to the intracellular domain^5,6^. The juxtamembrane domain, located intracellularly, contributes to receptor activation by facilitating proper orientation of the intracellular kinase domains and plays a role in allosteric regulation of EGFR activity. The intracellular tyrosine kinase domain, the catalytic core of EGFR, consists of an N-lobe and a C-lobe, with an ATP-binding site located between them. In its active conformation, the kinase domain forms an asymmetric dimer, where one kinase domain (activator) stimulates the catalytic activity of the other (receiver), crucial for EGFR-mediated phosphorylation of tyrosine residues. Finally, the C-terminal tail contains multiple tyrosine residues that, upon phosphorylation during receptor activation, serve as docking sites for various adaptor proteins, linking EGFR activation to intracellular signaling cascades^7^.

The identification of epidermal growth factor receptor (EGFR) mutations as oncogenic drivers has facilitated the development of targeted therapeutic interventions. EGFR tyrosine kinase inhibitors (TKIs), small molecule drugs, selectively bind to the ATP-binding domain of the EGFR kinase, thereby blocking its activity and inhibiting downstream signaling^8^. First-generation EGFR TKIs, including gefitinib and erlotinib, have demonstrated substantial clinical benefits in NSCLC patients harboring EGFR-activating mutations, resulting in improved progression-free survival and enhanced quality of life. However, the emergence of resistance to these first-generation TKIs has presented a significant clinical challenge. The most prevalent resistance mechanism is the development of the T790M mutation within the EGFR gene, which reduces the binding affinity of the TKIs. To circumvent this resistance, second-generation TKIs, such as afatinib and dacomitinib, were developed, exhibiting broader specificity and the capacity to inhibit the T790M-mutant EGFR^9^. More recently, third-generation TKIs, including osimertinib, have demonstrated remarkable efficacy in targeting both EGFR-activating mutations and the T790M resistance mutation. Consequently, osimertinib has become the standard of care for first-line treatment of EGFR-mutant NSCLC, demonstrating superior progression-free survival and overall survival compared to earlier generation TKIs^10,11^.

However, patients taking TKIs drugs ultimately develop further resistance against the used drugs^11^. For that reason, new TKIs have to be constantly developed to allow for an appropriate response. This study introduces DeepEGFR, a novel multi-class graph neural network (GNN) method designed for the precise identification of epidermal growth factor receptor (EGFR) inhibitors. A key distinguishing feature of DeepEGFR is its structure-independent protocol, which leverages Simplified Molecular Input Line Entry System (SMILES) notation as input, thereby predicting EGFR inhibition without requiring explicit three-dimensional structural information. DeepEGFR effectively integrates SMILES-based molecular graph representations and interpretable molecular fingerprints; specifically, Klekota-Roth and PubChem; within a cohesive architecture. This integration allows the model to capture complementary structural and substructural information proficiently. To the best of our knowledge, applying this dual-representation strategy within a Graph Neural Network (GNN) framework has not been previously utilized in classifying EGFR inhibitors. Consequently, this approach yields enhanced performance alongside greater mechanistic interpretability.

We show that DeepEGFR, by using an efficient structure representation, and advanced machine learning models, has a higher prediction quality than other tools. To enhance interpretability of our approach and to provide insights into the determinants of predicted activity, we applied a SHapley Additive exPlanations (SHAP) analysis, an explainable AI method, which enables the assessment of feature importance. As the used features correspond to substructures, the feature importance allows to identify key substructures contributing to inhibitory activity. We found that our identified substructures were also observed in known highly active, existing TKIs drugs. Furthermore, molecular docking studies demonstrated that these potential compounds bind at the same active site on the EGFR protein as FDA-approved drugs, strongly suggesting an analogous mechanism of action. This mechanistic similarity was further validated through molecular dynamics (MD) simulations, which were performed on the top five ranked compounds, revealing stable and sustained binding profiles indicative of robust interactions.

## Materials and methods

### Data curation

A dataset of compounds targeting the epidermal growth factor receptor (EGFR) was retrieved from the ChEMBL database (version 34, accessed December 2024), yielding an initial set of 35,310 bioactivity records associated with 11,634 unique chemical entities. In accordance with our previous work^12–14^, compounds were categorized based on their reported IC50 values: those with IC50 ≤ 1 µM were labeled as active, reflecting potent inhibition of EGFR; compounds with IC50 values between 2 and 9 µM were considered intermediate, indicating moderate activity; and those with IC50 ≥ 10 µM were deemed inactive, as they are unlikely to exert meaningful inhibitory effects on EGFR signaling. This classification scheme is consistent with criteria adopted in earlier pharmacological and cheminformatics analyses of kinase inhibitors^15–17^. Following data curation and filtering, the final dataset comprised 8,263 compounds, including 4,884 actives, 1,307 intermediates, and 2,072 inactives.

### Feature engineering

Molecular descriptors are numerical values that describe important features of compounds that are encoded within chemical structures and are used for subsequent model building. In this study, The PaDEL Descriptor software^18^ was used to compute 12 distinct molecular fingerprints from nine varied types as follows: AtomPairs 2D, CDK fingerprint, CDK extended, CDK graph only, E-state, Klekota-Roth, MACCS, PubChem and Substructure. The molecular descriptors were extracted using an in-house Python script. Of the twelve fingerprint types generated by PaDEL, we prioritized five (Klekota–Roth, PubChem, MACCS, EState, and Substructure) for SHAP interpretability due to their complementary strengths: Klekota–Roth and PubChem offer high structural coverage across diverse chemotypes; MACCS and Substructure capture well-defined functional groups common in kinase inhibitors; and EState encodes electronic and topological state information relevant to binding interactions. Focusing on these five enabled clear mechanistic insights (via SHAP feature attributions) while still covering the key structural and physicochemical determinants of EGFR inhibitor activity.

### QSAR modelling

This study adheres to the OECD guidelines^19^ for developing robust QSAR models, encompassing: (a) a clearly defined endpoint for the dataset, (b) an explicit learning algorithm, (c) well defined applicability domain (AD) for the QSAR model, (d) appropriate metrics for evaluating goodness-of-fit, robustness, and predictivity, and (e) mechanistic interpretation of the QSAR model. To address the latter point, the development of interpretable QSAR models, this research employs interpretable molecular fingerprints generated by the PaDEL-Descriptor software. Specifically, five out of the twelve available fingerprints (PubChem, Substructure, Substructure count, Klekota-Roth, and Klekota-Roth count) are inherently interpretable. Table 1 provides a comprehensive list of these fingerprints along with their respective descriptions.

### Protein and ligand preparation

The crystal structure of wild-type epidermal growth factor receptor (EGFR) was retrieved from the Protein Data Bank (PDB ID: 1M17)^20^. In preparation for molecular docking and dynamics, we removed co-crystallized ligand and associated crystal water molecules from the parent structure to ensure that the binding affinity of the screened drugs was not influenced by pre-bound molecules. The resultant EGFR structure was refined and prepared for subsequent analysis. To ensure the protein reaches its most stable conformational state, the structure was subjected to energy minimization after the ligand. SDF files of five FDA-approved EGFR inhibitors (afatinib, dacomitinib, osimertinib, gefitinib, and erlotinib) were obtained from the PubChem website. Open Babel was used to convert the SDF files to 3D structures, followed by energy minimization using the MMFF94 force field^21^. The inclusion of the EGFR structure and FDA-approved drugs was fundamental to our investigation, enabling precise molecular docking and the subsequent evaluation of potential EGFR inhibitors among these compounds.

### Molecular docking

AutoDock Vina (v 1.2.5)^22^ was used to dock the most common FDA-approved EGFR inhibitors into the crystallographic structure of the EGFR kinase domain (PDB ID 1M17). The protein was first prepared by assigning protonation states appropriate for pH 7.4 with PropKa, adding polar hydrogens, and applying Gasteiger charges; water molecules situated more than 5 Å from the ATP pocket were removed. Each ligand was energy-minimised with the MMFF94 force field and saved in PDBQT format. Blind docking was performed with a grid spacing of 1 Å and an exhaustiveness setting of 10, generating ten plausible poses for each ligand. To verify the protocol, erlotinib, the co-crystallised ligand of 1M17, was redocked. The leading pose reproduced the experimental orientation with an all-atom RMSD of 0.84 Å, supporting the accuracy of the docking parameters. For every inhibitor, the pose with the most favourable predicted binding free energy (ΔG, kcal mol⁻¹) and the clearest interaction pattern was chosen for further study. Contact analysis proceeded along two lines: (i) hydrogen-bond and hydrophobic interaction counts were extracted from Vina output with a Biopython-based script, and (ii) LigPlot + v2.2^18^ was used to generate 2D presentation of ligand-protein complexes. These visualisations provided a concise comparison of how each drug engages the EGFR pocket and formed the basis for subsequent molecular-dynamics stability evaluation^23^.

### Molecular dynamics simulations

All-atom MD simulations play a crucial role in drug discovery, particularly in understanding the dynamic behavior of proteins and their interactions with ligands. The docked complexes obtained from AutoDock Vina were subjected to molecular dynamics (MD) simulations using GROMACS 2023.3^24^. The protein was parameterized using the CHARM force field, and the ligands were parameterized using the SwissParm website^25^. Each complex was solvated in an orthorhombic box of TIP3P water molecules, and Na + and Cl- ions were added to neutralize the system. Prior to the simulations, an energy minimization step was executed to remove unfavorable interactions. This involved 1,500 steps of steepest descent energy minimization to optimize the system’s stability. This condition ensures that the protein does not interact with itself. With positional restrictions on ligand atoms and protein heavy atoms, the energy of solvated systems is then minimized. The system is equilibrated for 10 ns under constant volume (NVT) then run for 10 ns at constant pressure and temperature (NPT). Electrostatic interactions are calculated using the particle mesh Ewald method A cutoff radius of 10 Å is applied for both the electrostatic interactions and the Van der Waals interactions. The P-LINCS algorithm is employed to constrain all covalent hydrogen bonds. Pressure is kept at 1 bar using the Parrinello-Rahman pressure coupling and temperature is kept at 298 K using velocity rescaling with a stochastic term. The time constants for the temperature and pressure couplings to the bath were 0.1 and 2 ps, respectively. The trajectory was saved every 100 picosecond, and a total length of the MD simulation was 100 ns. Additionally, we harnessed the molecular mechanics/generalized born surface area (MM/GBSA) method to evaluate the binding energy of the dynamic receptor–ligand complex. The intricate calculations were executed using the gmx_MMPBSA tool^26^, based on MM-PBSA.py^27^ in AmeberTools23. To accommodate computational constraints, we selectively employed the MD trajectory from the final 20 ns (80–100 ns) of the simulation for a binding energy assessment.

### Graph neural network (GNN) method

**Fig. 1:**
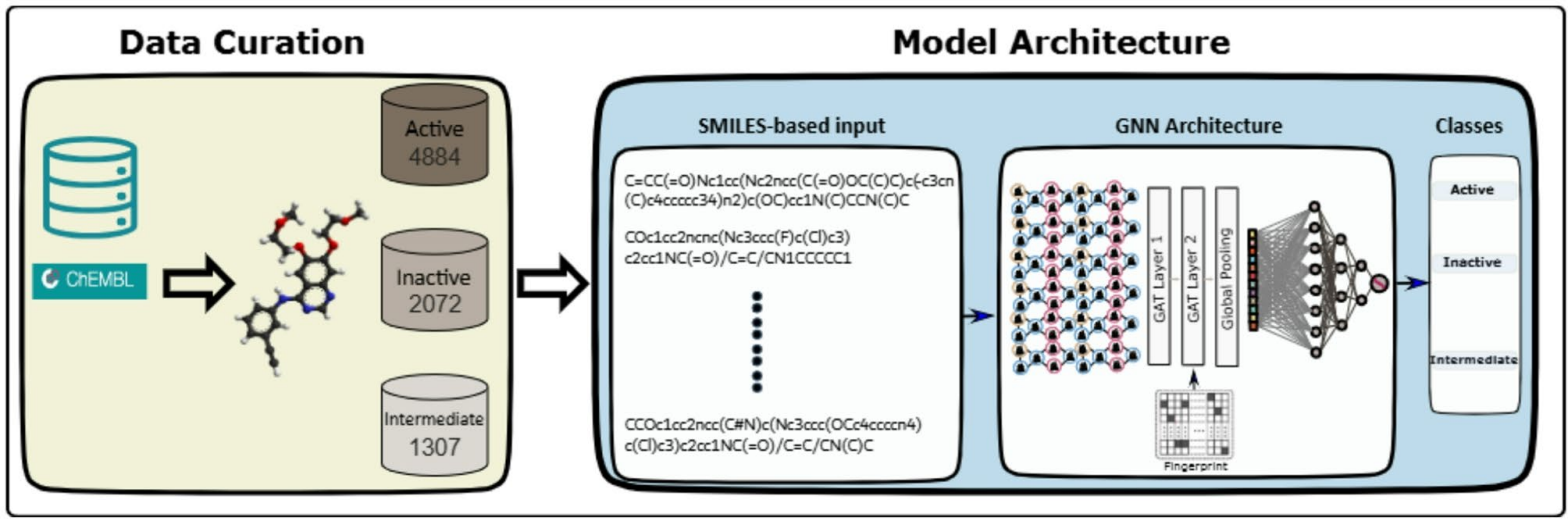
Overview of the DeepEGFR model architecture and data curation workflow. It illustrates the DeepEGFR framework, showcasing the integration of SMILES-based input and molecular fingerprints for classifying compounds into three activity classes: Active, Inactive, and Intermediate. The architecture includes Graph Attention Layers (GAT Layer 1 and GAT Layer 2), followed by a global pooling layer to aggregate node features. The processed data is then fed into the Graph Neural Network (GNN) for activity classification. Additionally, the figure highlights the data curation process, including the distribution of compounds across activity classes.

In this study, we introduce a novel approach called DeepEGFR, which leverages the complementary information encoded in both Simplified Molecular Input Line Entry System (SMILES) strings and molecular fingerprint matrices to comprehensively understand the activity of Epidermal Growth Factor Receptor (EGFR) inhibitors.We employed a parallel explanatory design, initially analysing molecules represented as SMILES strings and subsequently examining molecular fingerprint matrices to further validate and elaborate upon the findings. DeepEGFR is developed as a multi-class graph neural network (MC-GNN) model, which classifies molecules into three activity categories: Active, Inactive, or Intermediate. The overall architecture is outlined in Fig. 1. To enhance the model’s ability to capture important structural and property information from molecular graphs, we incorporated graph-based node and graph edge features. These features, inspired by prior work (Li et al., 2018), include (1) Node Features: atom type, atomic number, and formal charge; and (2) Edge Features: bond type and bond order. The graph is initially processed by an embedding layer composed of three fully connected layers with 128, 64, and 32 neurons, respectively. These are followed by two graph attention network (GAT) layers, which update node embeddings using attention-weighted message passing to prioritize chemically relevant neighbors. In parallel, the fingerprint matrix is encoded and concatenated with the final graph-level representation, allowing the model to integrate both topological and substructural information. The combined feature representation is passed through a dense classification layer with three output neurons. The fitness function used to guide model training was categorical cross-entropy, which is appropriate for multi-class classification tasks and measures the divergence between predicted probabilities and true class labels.

In a post processing step, we explored the distribution of key physicochemical properties, such as molecular weight (MW), logP, and Lipinski’s Rule of Five (Ro5)^28^ descriptors, across active, inactive, and intermediate compounds. This analysis provides the insights into the chemical diversity of the dataset enabling us to make a final set of classified compounds with likely EGFR activity and drug-like properties after processing by the DeepEGFR model. By refining the final dataset through this approach, we ensure that the model focuses on compounds with a higher likelihood of exhibiting biological activity, thereby enhancing predictive performance.

For the model training and optimization, we applied a robust grid search^29^ to fine-tune hyperparameters. Key hyperparameters, such as the number of GNN layers [1–3], the number of neurons in each layer [64, 128, 256], the learning rate [0.0001 to 0.01], and the batch size [32, 64, 128], were optimized based on performance on a training and validation set. To ensure robust generalization and prevent data leakage, we employed a scaffold-based splitting strategy using the Bemis-Murcko scaffold decomposition implemented via RDKit. Compounds were grouped by core structural frameworks and split such that 80% of scaffolds were used for training, 10% for validation, and 10% for testing. This ensures that structurally similar compounds do not appear across sets and allows a more realistic evaluation of model performance. The final model was evaluated on a separate test set to confirm its effectiveness. During hyperparameter tuning, the F1-score for each activity class (Active, Inactive, and Intermediate) was closely monitored across all 50 training epochs. Supplementary Figure S1 presents the training and testing F1-scores for each class across the training epochs, demonstrating excellent performance for all three classes, achieving approximately 94% F1-score for both training and test sets. The fitness function used to guide model training was categorical cross-entropy, which is appropriate for multi-class classification tasks and measures the divergence between predicted probabilities and true class labels. This systematic tuning ensured that DeepEGFR’s architecture was not arbitrarily selected, but optimized for generalization and balanced class performance.

The architecture of DeepEGFR starts with graph embedding, which generates dense vector representations of the input molecular graphs. This layer consists of three fully connected (dense) layers with 128, 64, and 32 neurons, respectively. The first two layers utilize Rectified Linear Unit (ReLU) activation functions, while the final layer employs a softmax activation function. Multiple GNN layers subsequently aggregate node features and update the graph embeddings. Each GNN layer is structured identically to the embedding layer, consisting of three fully connected layers with 128, 64, and 32 neurons, using ReLU activation in the first two layers and softmax in the last.

Finally, the classification predicts the probability distribution of the input molecule within the three activity classes: Active, Inactive, or Intermediate. The classifier consists of a single fully connected (dense) layer with 3 neurons, followed by a softmax activation function.

## Results and discussion

### Chemical space analysis

**Fig. 2:**
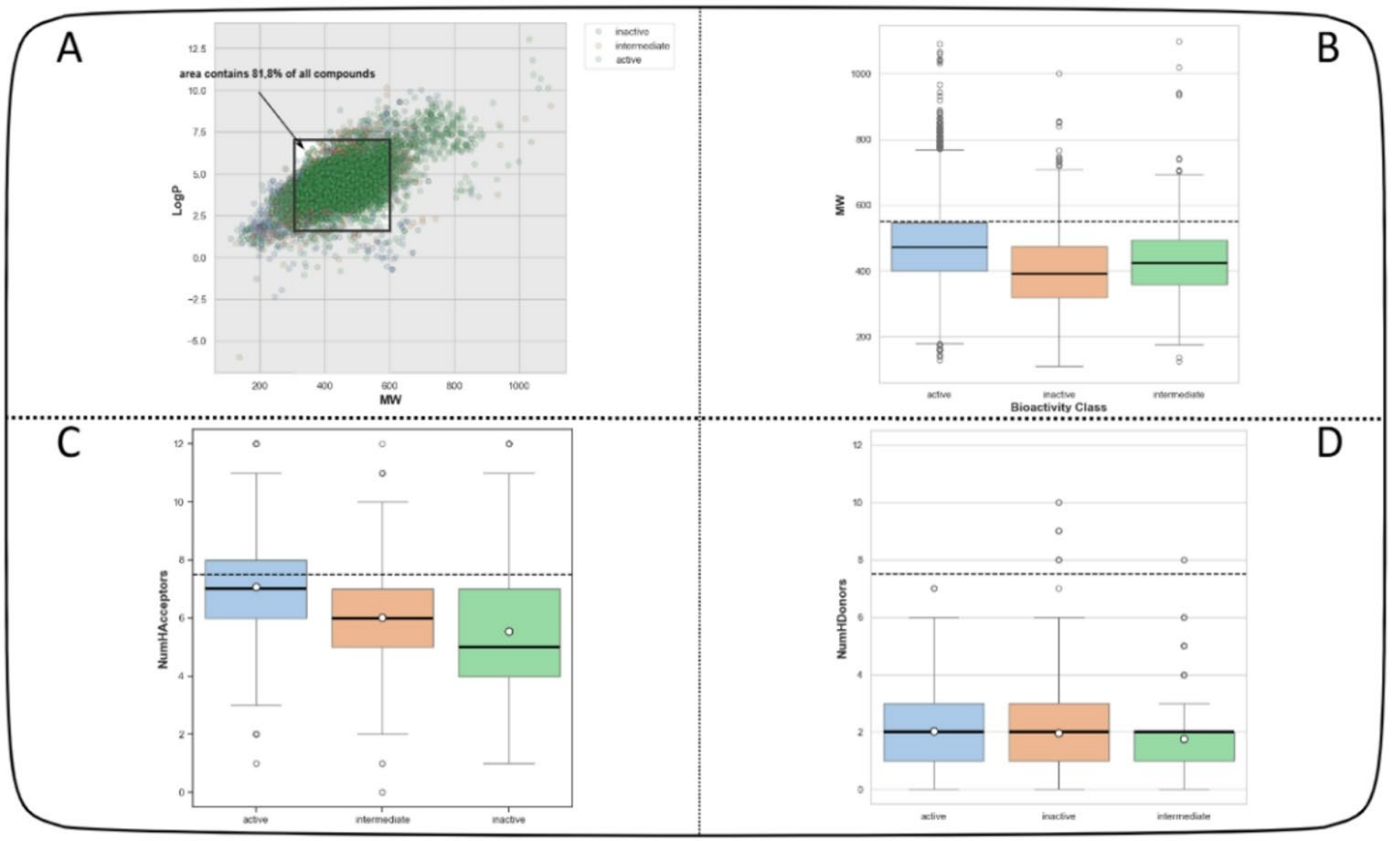
Chemical space analysis of EGFR inhibitors (**A**) Representation of the correlation between molecular weight (MW) and the Ghose-Crippen-Viswanadhan octanol-water partition coefficient (LogP). Light green are active while as light blue and orange are inactive and intermediate compounds. (**B**,** C** and ** D**) Box plots comparing active intermediate and inactive compounds based on Lipinski’s Rule of Five descriptors. The dashed lines denote the threshold for drug-like properties: molecular weight (MW) < 600, Ghose-Crippen-Viswanadhan octanol-water partition coefficient (LogP) < 7, number of hydrogen bond donors (NumHDonors), number of hydrogen bond acceptors (NumHAcceptors) < 10. Circles indicate the average values, while asterisks mark statistically significant differences between the two groups (p-value < 0.05).

We conducted a chemical space analysis to identify the underlying chemical characteristics between active, inactive and intermediate compounds. The relationship between the Ghose-Crippen-Viswanadhan octanol-water partition coefficient (LogP)^30^ and molecular weight (MW) was analyzed to explore the distribution of active, as well as their pIC50 values, within the context of LogP and MW as depicted in Fig. 1A. LogP, a physicochemical indicative of lipophilic properties, is crucial for assessing a compound’s ability to permeate the cell membrane and its drug-likeness^31^. Subsequently, we examined the chemical properties according to Lipinski’s rule-of-five (Ro5) descriptors, as illustrated in Fig. 2^32^. This rule, derived from the characteristics of orally administered drugs, proposes four parameters indicative of drug-likeness: MW < 500 kDa, LogP < 5, the number of hydrogen bond donors (NumHDonors) < 5, and the number of hydrogen bond acceptors (NumHAcceptors) < 10. compounds exceeding the specified range for any two parameters tend to exhibit poor absorption or permeability and a higher likelihood of failure in drug development. Figure 2 reveals that the distribution of active, inactive and intermediate compounds largely overlaps, predominantly falling within a LogP range of 2–7 and an MW range of 300–600. Additionally, the Ro5 analysis (dashed line, Fig. 2C and D and statistical analysis revealed a discrepancy in adherence within the active compound group. Specifically, while some active compounds followed Ro5 guidelines for molecular weight (MW) and LogP, others did not. In contrast, all three categories of compounds adhered to Ro5 guidelines for NumHDonors and NumHAcceptors.

### Performance evaluation of DeepEGFR: a comparative analysis with baseline models

**Fig. 3:**
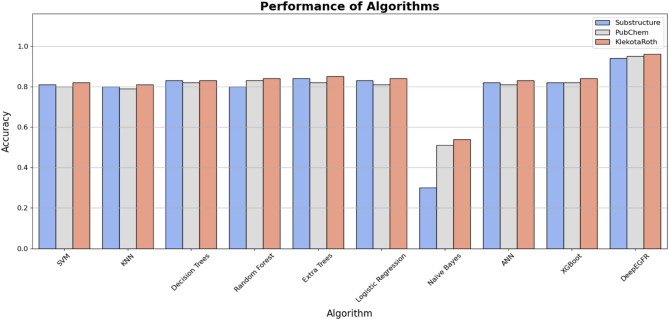
Comparative performance of DeepEGFR against baseline algorithms (SVM, KNN, Decision Trees, Random Forest, Extra Trees, Logistic Regression, Naïve Bayes, ANN, and XGBoost) across three molecular representations: Substructure-based features, PubChem fingerprints, and Klekota-Roth fingerprints. DeepEGFR consistently outperformed all other methods, achieving the highest accuracy across all feature types, demonstrating its superior ability to classify EGFR inhibitor activity (Active, Inactive, Intermediate).

We compared the proposed method DeepEGFR with different types of machine learning (ML) approaches, including Support Vector Machines, Naive Bayes, K-nearest Neighbours, Fully Connected Neural Networks, Decision Trees, Random Forest, and extra trees. All baseline models were implemented using scikit-learn (v1.3.0) and trained using the same curated dataset. To ensure a fair comparison, each model was trained using the same scaffold-based train/validation/test splits, and feature inputs (PubChem and Klekota-Roth fingerprints) identical to those used by DeepEGF. Hyperparameters for each model were optimized via grid search, and best-performing configurations. The evaluation metrics encompassed accuracy for three distinct molecular representations: Substructure-based features, PubChem fingerprints, and Klekota-Roth fingerprints. Figure 3 illustrates that the DeepEGFR model significantly outperformed all other algorithms across every feature representation. Specifically, it achieved near-perfect classification accuracy, demonstrating its superior ability to distinguish between molecular activity classes (Active, Inactive, and Intermediate). Among the baseline models, Random Forest and ANN exhibited moderate performance, but they could not match the robust accuracy of DeepEGFR. This result underscores the effectiveness of graph neural networks in capturing complex molecular relationships and their applicability in EGFR inhibitor classification. The comparison also revealed the importance of feature representation in model performance. In addition to conducting model benchmarking, we performed ablation experiments to evaluate the contribution of molecular fingerprints. Interestingly, when the Klekota-Roth and PubChem features were excluded and DeepEGFR was trained exclusively on graph-based representations, the average F1-score exhibited a decline across various activity classes. These results underscore the importance of integrating both graph and fingerprint-based descriptors in enhancing DeepEGFR’s performance, thereby affirming the efficacy of a dual-representation framework (see supplementary table Table S2).

DeepEGFR consistently demonstrated high accuracy regardless of the feature type, highlighting its adaptability and capacity to extract meaningful information from structural and fingerprint-based molecular representations.

In contrast, traditional machine learning models showed greater sensitivity to the choice of input features, with their accuracy varying significantly across the three feature sets. In addition to overall accuracy, the DeepEGFR model achieved a balanced classification performance across all three activity classes (Active, Inactive, Intermediate). As depicted in supplementary Figure S1, the F1-scores for all classes remained stable and high throughout training, reaching approximately 94% for both training and test datasets. This consistent performance indicates the model’s ability to generalize to unseen data while maintaining class-specific precision and recall.

The DeepEGFR approach substantially improves over traditional methods for classifying EGFR inhibitors. While conventional models rely heavily on engineered features or molecular fingerprints, the graph-based approach of DeepEGFR offers a more holistic understanding of molecular interactions. This is particularly evident in its ability to process SMILES strings and integrate graph embeddings, leading to more accurate predictions and meaningful insights into molecular activity.

### High value feature detection for DeepEGFR classifier

To understand deeper insights into the decision-making process of DeepEGFR, we conducted an SHAP analysis to identify high value features to identify the molecular descriptors that significantly influenced the model’s classification of EGFR inhibitors. Using SHAP values provides a consistent, model-agnostic measure of feature contribution, allowing us to interpret how individual fingerprint features impacted the predicted probability for each activity class. We used the SHAP Python library (version 0.41.0) and implemented a model-specific DeepExplainer to compute SHAP values, as DeepEGFR is a deep learning-based model. SHAP values were computed for a random, stratified sample of 500 compounds drawn from the validation set, ensuring balanced class representation while keeping computation manageable. We calculated mean absolute SHAP values across this representative sample to rank features by their overall contribution. The analysis was performed on the final trained classifier using a representative subset of the validation data to balance computational cost and interpretability. We extracted the top 20 most important features from SHAP values were calculated for both the Klekota-Roth and PubChem molecular fingerprint matrices, providing a comprehensive understanding of structural properties contributing to inhibitory activity. The mechanistic of these substructures were summarized in Table 2 and supplementary Table S1.

**Fig. 4:**
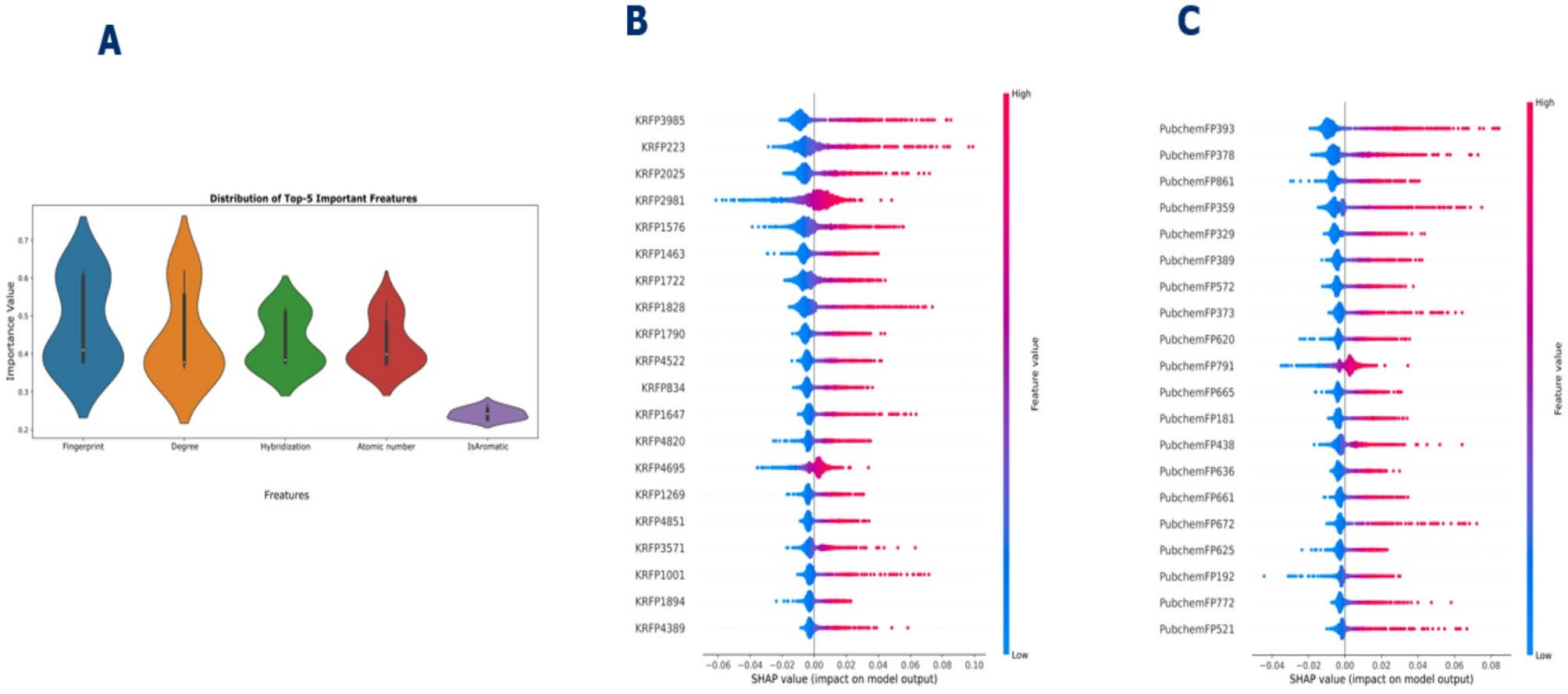
(**A**) Overall feature importance rankingFeature importance analysis based on the SHAP method for DeepEGFR from Kleokota-Roth and Pubchem (**B**,** C**). These SHAP values represent the directionality features where positive and negative values influences the prediction towards positive and negative samples.

We extracted the top 5 most influential features, which play a dominant role in distinguishing between Active, Inactive, and Intermediate inhibitors. The results of this analysis are illustrated in Fig. 4A, B, and C, where Fig. 4A presents the overall feature importance ranking. In contrast, Fig. 4B and C display SHAP values of the top 20 features derived from the Klekota-Roth and PubChem fingerprints, respectively. The feature importance analysis revealed that critical molecular descriptors included hydrophobic interactions, electrostatic properties, and hydrogen bond donor/acceptor patterns, all of which are essential for ligand-protein binding affinity.

The 5-top ranked feature included hydrophobic groups, aromatic rings, and key functional moieties that enhance binding interactions within the EGFR active site. These findings suggest that compounds exhibiting similar molecular patterns may possess strong inhibitory potential. The Klekota-Roth features predominantly captured substructural patterns and functional groups associated with bioactivity. In contrast, the PubChem features provided a broader representation of atomic connectivity and chemical fragments relevant to EGFR inhibition. Furthermore, many of these identified features align with the well-characterized molecular properties of FDA-approved EGFR inhibitors, such as Afatinib, Gefitinib, Osimertinib, Dacomitinib, and Erlotinib, further validating the reliability of DeepEGFR’s feature selection. Interestingly, our findings align with previously identified substructures present in the majority of FDA-approved EGFR inhibitors. These substructures demonstrate molecular characteristics indicative of potent inhibitory capabilities, suggesting their potential utility as novel scaffolds for drug development. The presence of these substructures highlights DeepEGFR’s ability to highlight the important molecular features that could contribute to the next generation of EGFR-targeting drugs.

### Mechanistic significance of the Kleokota-Roth and PubChem fingerprints

The top-ranked features identified through SHAP analysis (Table 2) were further examined to explore their mechanistic significance in EGFR inhibition. By analyzing the molecular substructures captured by the Klekota-Roth and PubChem fingerprints, we aimed to understand how these features contribute to ligand-protein interactions within the EGFR kinase domain.

Thus, we investigated the most significant molecular descriptors identified by DeepEGFR, focusing on the top 10 ranked fingerprints from both the Klekota-Roth and PubChem fingerprints. These descriptors highlight key substructures that contribute to the inhibitory activity against EGFR, offering insights into how specific chemical features interact with the EGFR kinase domain. The mechanistic significance of these fingerprints is discussed below, with detailed contributions of their substructures toward EGFR inhibition presented in Table 2 and supplementary Table S1.

The Klekota-Roth fingerprints (KRPF) capture substructural motifs commonly associated with bioactivity, providing detailed insights into functional groups and molecular frameworks relevant to EGFR inhibition. One of the most prominent features identified is 4-Vinylphenol (KRFP1463), a phenolic derivative with a para-hydroxy group. Its vinyl group facilitates covalent modification of critical cysteine residues, such as Cys797, within the ATP-binding pocket of EGFR. This covalent interaction leads to irreversible inhibition, effectively shutting down EGFR signaling pathways crucial for cancer cell proliferation^33,34^. Another significant feature, 1,1-Ethanediol (KRFP25), despite its simple structure, contains hydroxyl groups that enable hydrogen bonding interactions with key residues in the EGFR active site. This substructure has been shown to specifically interact with the EGFR T790M mutant which accounts to 40–55% drug resistance for the first-generation EGFR kinase inhibitors in the NSCLC^35^. These interactions enhance the molecule’s binding affinity and therapeutic potential. Similarly, Quinoline (KRFP1828), a well-established pharmacophore in medicinal chemistry, contributes through its heteroaromatic scaffold, promoting π-π stacking and hydrogen bonding. Quinoline-based compounds were also shown to have promising inhibitory activity against EGFR^36–39^. Moreover, this rigid, planar structure enhances binding stability, as observed in FDA-approved EGFR inhibitors like Gefitinib and Erlotinib. Additionally, features such as 5,5-Dimethyl-cyclohex-2-enone-3-ol (KRFP3459) and 1-Bromo-3-chlorobenzene (KRFP1790) highlight the importance of hydrophobic interactions and halogen bonding. Halogen atoms, particularly bromine and chlorine, enhance lipophilicity and facilitate unique non-covalent interactions with electron-rich regions in EGFR. Previous study supporting halogen bonding manifest into water mediated hydrogen bonds leading to Kinase inhibitory activity in EGFR^36,40,41^. Lastly, the presence of Pyrimidine (KRFP2025), a scaffold that mimics the adenine ring of ATP, further emphasizes the importance of competitive inhibition at the ATP-binding site. Pyrimidine derivatives block phosphorylation and stabilize EGFR in its inactive conformation, enhancing therapeutic efficacy^42,43^. Compounds like Phenyl(pyridazin-4-yl) methanone (KRFP1576) and Phenylethyl Alcohol (KRFP4820) combine aromatic systems with polar functional groups, offering a balance of hydrophobic and hydrogen-bonding interactions that optimize binding affinity^44,45^.

Compared to Klekota-Roth fingerprints, PubChem fingerprints capture a broader range of chemical functionalities and atomic connectivity patterns, providing complementary insights into molecular determinants of EGFR inhibition. The top-ranked feature, 2-Bromoaniline (PubChemFP772), shares mechanistic similarities with halogenated Klekota-Roth features, where the bromine atom and amino group participate in halogen bonding and hydrogen bonding, respectively. These interactions target the ATP-binding pocket, disrupting kinase activity and downstream signaling^46^. Another key feature is 1,2-Dimethylcyclopentane (PubChemFP861), which contributes to hydrophobic interactions with non-polar residues in EGFR. Although not a traditional pharmacophore in kinase inhibitors, its rigid cyclic structure offers a hydrophobic scaffold for further functionalization, enhancing binding affinity. The importance of amine-containing structures is reflected in features like 2-Propanimine (PubChemFP568) and N-butylamine (PubChemFP665), which act as hydrogen bond donors, facilitating critical interactions with polar residues within the active site. Next, the identification of unsaturated non-aromatic rings such as Cyclohexene (PubChemFP189) underscores the role of flexible hydrophobic scaffolds in EGFR inhibition. These structures support van der Waals interactions and can be optimized for better receptor binding^42^. Similarly, Acrolein (PubChemFP672), despite its inherent reactivity, forms covalent bonds with cysteine residues, leading to potent but potentially toxic inhibition. However, its activity can be modulated when incorporated into larger, more stable inhibitor frameworks^37^. Finally, compounds like Ethoxymethanol (PubChemFP661) and p-Phenylenediamine (PubChemFP728) highlight the importance of polar functional groups in enhancing water solubility and bioavailability while maintaining strong binding interactions with EGFR. The combination of hydrophilic and hydrophobic features in these fingerprints provides a blueprint for designing novel inhibitors with optimized pharmacokinetic properties.

The mechanistic insights gained from the analysis of these fingerprints are supported by molecular dynamics (MD) and docking simulations. These analyses confirmed that most of the top-ranked features identified by DeepEGFR engage in key interactions with critical residues in the EGFR active site, such as Lys745, a conserved residue within the ATP-binding site of the kinase family. Additionally, the Top2 inhibitor demonstrates stable interactions with Leu792, Met793, and Pro794; residues that flank the gatekeeper residue Thr790; highlighting its potential to modulate EGFR activity and possibly overcome resistance associated with T790M mutations. Moreover, the fingerprints found be important by DeepEGFR model revealed potential new substructures not yet present in FDA-approved EGFR inhibitors, suggesting potential new avenues for drug discovery. These substructures exhibit promising binding characteristics, as demonstrated by their high docking scores and favorable interaction profiles. Moreover, the fingerprint revealed potential new substructures not yet present in FDA-approved EGFR inhibitors, suggesting potential new avenues for drug discovery. These substructures exhibit promising binding characteristics, as demonstrated by their high docking scores and favorable interaction profiles.

### Docking validation of FDA-approved EGFR inhibitors

**Fig. 5:**
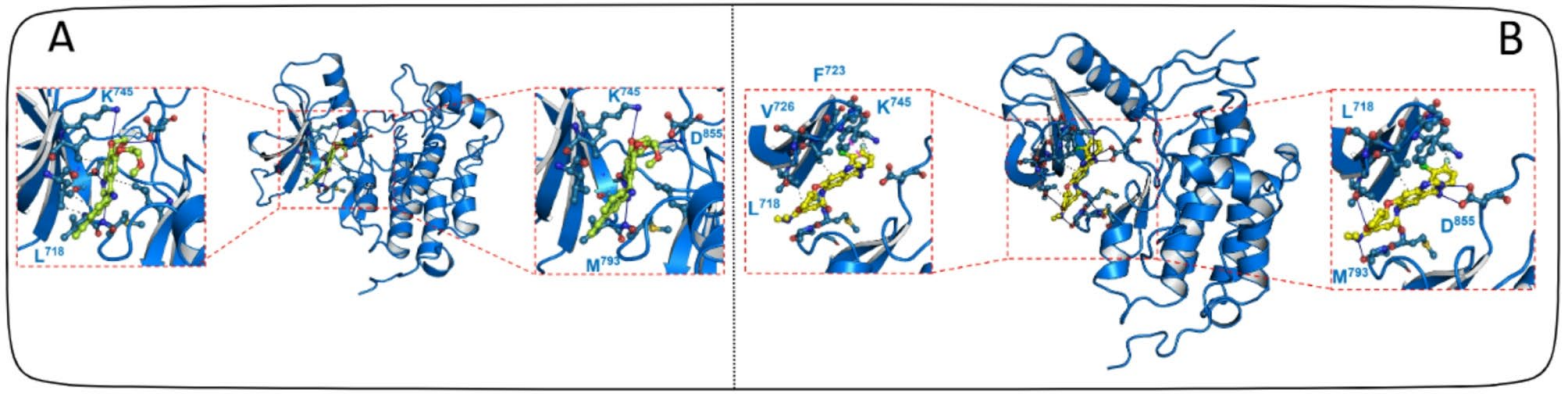
Molecular docking of FDA-approved drugs (**A**) Binding interaction of Erlotinib and (**B**) Afatinib with wild type EGFR (1M17) and the ligand is colored in orange (Erlotinib) and green (Afatinib) while protein is in blue. The interactions are shown by dotted line between residues and ligands, pi-pi interactions are shown in orange dotted line while as hydrogen bonds and hydrophobic bonds are shown in solid and dotted grey lines, respectively.

To elucidate the mechanisms underlying ligand-protein interactions, we obtained all 47 FDA-approved drugs targeting EGFR from the ChEMBL database and performed molecular docking into the kinase domain of EGFR. The docking results were validated by comparing them with the co-crystal structure of Erlotinib (PDB ID: 1M17). Initially, the inhibitor was removed from the complex and subsequently redocked to confirm its binding to the same active site. This validation procedure reinforced the accuracy and reliability of the docking methodology. Due to the limited availability of co-crystal structures for FDA-approved EGFR inhibitors, the remaining inhibitors were assessed in comparison to this reference structure. Generally, lower docking scores of ligands correlated with higher binding affinity to the receptor. Although most inhibitors exhibited strong binding affinity to the kinase domain, only the most promising candidates were selected for further analysis. The molecular docking scores of EGFR in complex with FDA-approved drugs are presented in Figure S2. Subsequently, the most promising inhibitors, including CHEMBL553 (Erlotinib)^47^, CHEMBL3353410 (Osimertinib)^48^, CHEMBL939 (Gefitinib)^49^, CHEMBL1173655 (Afatinib)^50^, and CHEMBL2110732 (Dacomitinib)^51^, were chosen for further investigation and molecular dynamics (MD) simulations. Erlotinib is a targeted cancer therapy drug primarily used in the treatment of non-small cell lung cancer (NSCLC) and pancreatic cancer. It functions as an inhibitor of the epidermal growth factor receptor (EGFR) tyrosine kinase, thereby disrupting signaling pathways that promote tumor growth and survival. The amine group forms the hydrophobic interaction with the hinge region of the EGFR while as ethynylphenyl and bis(2-methylpropoxy) groups forms hydrophobic interaction with surrounding amino acids especially Leu858 and Met769 as shown in the Fig. 5A. Besides that, it also forms hydrogen bonds with Lys745, Asp855 and Met793^52^. Similarly, Osimertinib, specifically designed to target both sensitizing EGFR (such as exon 19 deletions and L858R) and the T790M resistance mutation commonly associated with acquired resistance. The efficacy of osimertinib is largely attributed to its precise interactions with key residues within the EGFR kinase domain, facilitating potent and selective inhibition^53^. As shown in figure, it forms hydrogen bonds with Lys745 and Arg841 while as with Phe723, forms pi-pi stacking. Furthermore, it also forms salt bridges with Asp837 and Asp855. To further stabilize that interaction, it forms hydrophobic interactions with Cys797, Leu714, Leu844 and Leu718 as shown in Fig. 5B. Overall, in our docking, we identified all the key residues that interacted with the inhibitor and blocks its functional activity (supplementary Figure S3).

### Mechanistic insight of inhibition through MD simulation

**Fig. 6:**
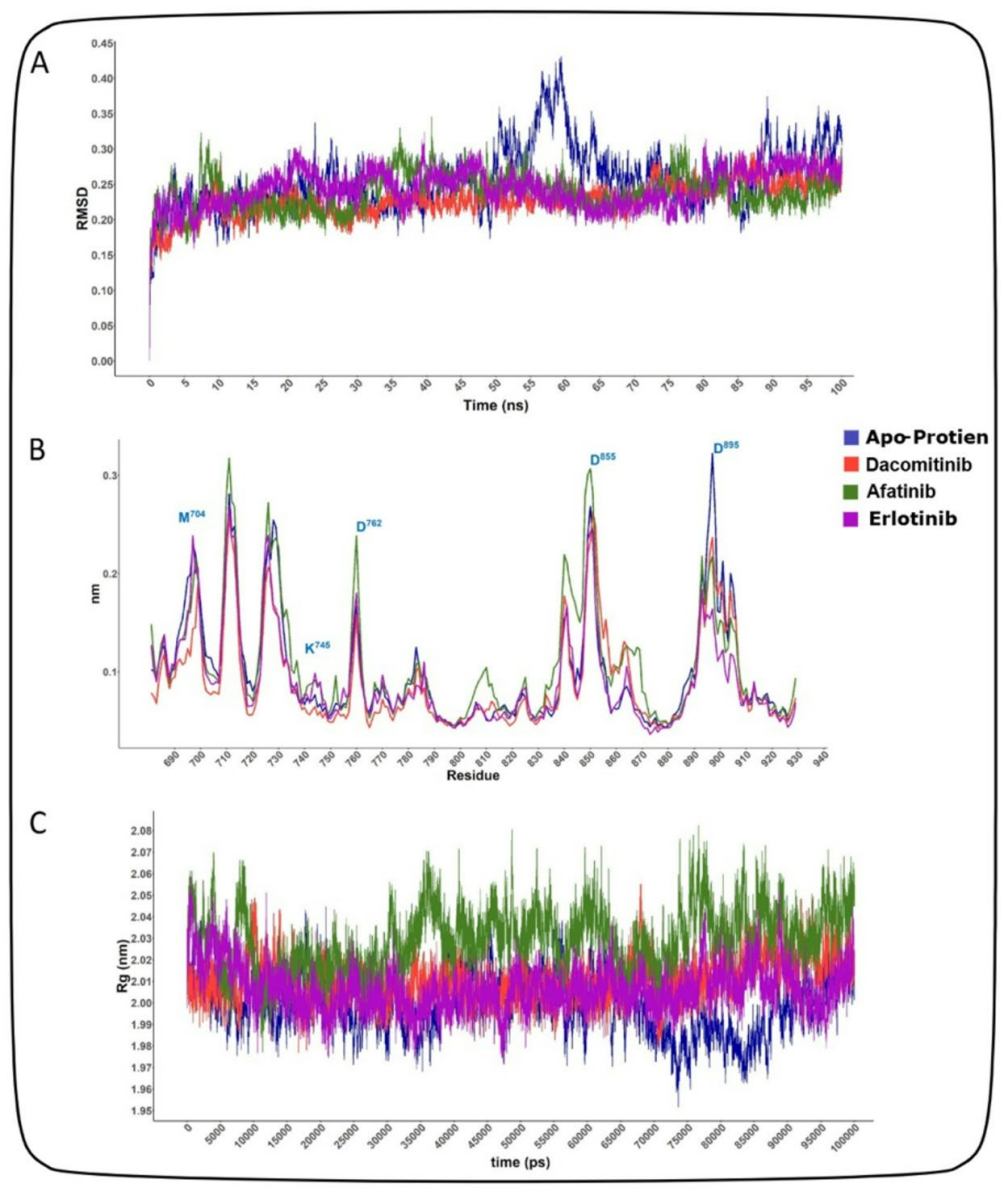
RMSD, RMSF, radius of gyration value of apo-protein of the selected four FDA-approved drugs in the complex with EGFR protein. In molecular dynamic simulation, (**A**) protein RMSD analysis, (**B**) RMSF value analysis (**C**), and radius of gyration, were analyzed for selected three compounds at 100 ns. The simulation was conducted in GROMACS and colors indicated FDA approved drugs, i.e., apo-protein (blue), Afatinib (green), Erlotinib (purple), Dacamitinib (red) and Osimertinib(blue).

To further validate the stability of the docked complexes, molecular dynamics (MD) simulations were conducted over a period of 100 ns. The root-mean-square deviation (RMSD) of the EGFR protein backbone was analyzed to assess the stability of the EGFR-drug complexes throughout the molecular dynamics simulations. As illustrated in Fig. 5A, all EGFR-drug complexes achieved equilibrium after approximately 20 nanoseconds (ns) of simulation time, exhibiting average RMSD values ranging from ~ 1.5 Å to ~ 2.0 Å. These consistent and low RMSD values indicate that the binding conformations of the FDA-approved drugs are stable within the EGFR active site over the course of the simulation. In contrast, the EGFR protein in the absence of any inhibitor, demonstrated significant instability, with RMSD values fluctuating beyond acceptable limits throughout the simulation period (depicted in Fig. 6A). This lack of stability in the apo form highlights the stabilizing effect conferred by the drug binding, underscoring the potential efficacy of the FDA-approved drugs in maintaining EGFR structural integrity. The root-mean-square fluctuation (RMSF) analysis highlighted that the binding site residues exhibited low flexibility, further supporting the stability of the drug-protein interactions (Fig. 6B). Throughout the MD simulations, the key interactions identified during docking remained stable. For instance, the hydrogen bond between Erlotinib and M793 was maintained for > 90% of the simulation time, while the hydrophobic interactions with L788 and V726 were consistently observed. The radius of gyration (Rg) analysis indicated no significant structural changes in EGFR upon drug binding, with Rg values remaining stable at ~ 22.5 Å (4 C). As shown in Fig. 6C, our MD simulation highlighted the stabilizing effects of Afatinib, Dacomitinib, and Erlotinib on the EGFR tyrosine kinase domain, with irreversible inhibitors providing the most significant stabilization. The apo form exhibited greater flexibility and conformational sampling, reflecting its intrinsic dynamics in the absence of a ligand. These results provided valuable insights into the mechanisms of inhibition and guide further drug design efforts.

**Fig. 7:**
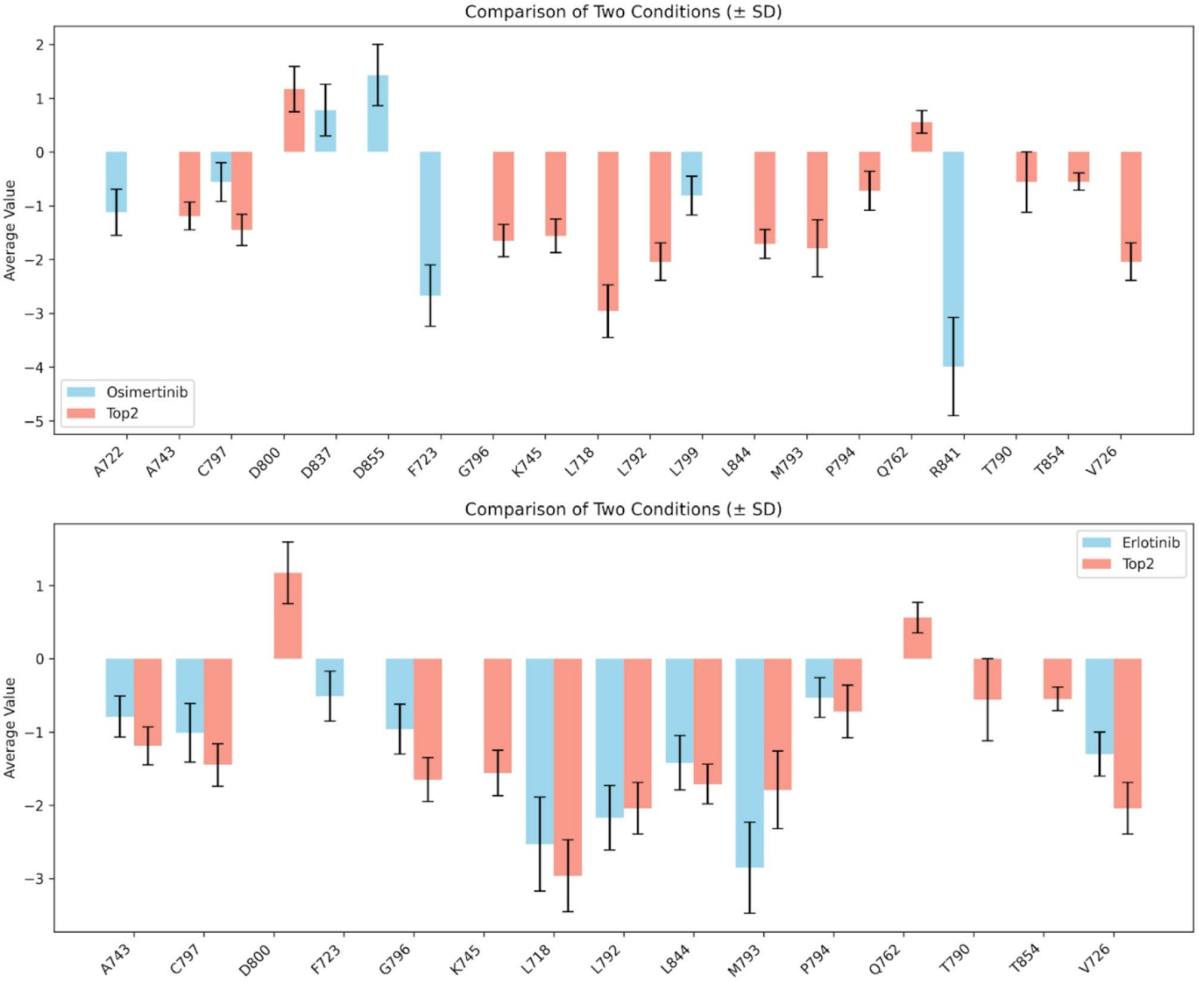
Per-Residue Energy Decomposition Comparison: (**A**) Osimertinib and Top2. This figure displays the per-residue energy decomposition for the binding of Afatinib and Potent under Investigated Inhibitor Top2 (red bars) to EGFR. The y-axis represents the energy contribution (kJ/mol) of each EGFR residue (x-axis) to the total binding free energy. Negative values indicate favorable binding contributions, while positive values indicate unfavorable contributions. (**B**) Erlotinib and Top2. This figure displays the per-residue energy decomposition for the binding of Osimertinib (blue bars) and Top2 (red bars) to EGFR. The y-axis represents the energy contribution (kJ/mol) of each EGFR residue (x-axis) to the total binding free energy. Negative values indicate favorable binding contributions, while positive values indicate unfavorable contributions.

Using the MM/GBSA approach, the binding free energies between EGFR (wild-type and mutants) and various ligands were calculated following molecular dynamics simulations. Notably, the underexplored compounds; Top2 (− 35.55 kcal/mol) and Top4 (− 24.18 kcal/mol); demonstrated binding affinities comparable to or exceeding those of several FDA-approved inhibitors, including Afatinib (− 32.75 kcal/mol), Gefitinib (− 29.61 kcal/mol), Erlotinib (− 26.43 kcal/mol), Osimertinib (− 11.97 kcal/mol), and Dacomitinib as shown in Table S3. To validate our computational approach, we compared the predicted binding energies of these compounds with their experimentally reported affinities. As shown in Table S5, the calculated values show strong concordance with the experimental data, thereby supporting the robustness and predictive reliability of our computational approach. This consistency between calculated and experimental binding energetics underscores the utility of molecular dynamics-based methods for accurate affinity prediction in drug discovery efforts targeting EGFR^54–56^. Importantly, Top2 retained high binding affinity across key EGFR resistance mutations; T790M, L858R, and C797S; suggesting its potential to overcome common resistance mechanisms (Table S4, Fig. S4–S8). Furthermore, per-residue energy decomposition analyses revealed that Top2 and Top4 contributed more favorable interactions from a larger number of residues within the EGFR binding pocket compared to Afatinib and Osimertinib (Fig. 7A and B). These results suggest that Top2, in particular, forms a broader and more stable interaction network, positioning it as a promising lead compound for the development of next-generation EGFR-targeted therapies.

### Detection of underexplored anti-EGFR compounds

**Fig. 8:**
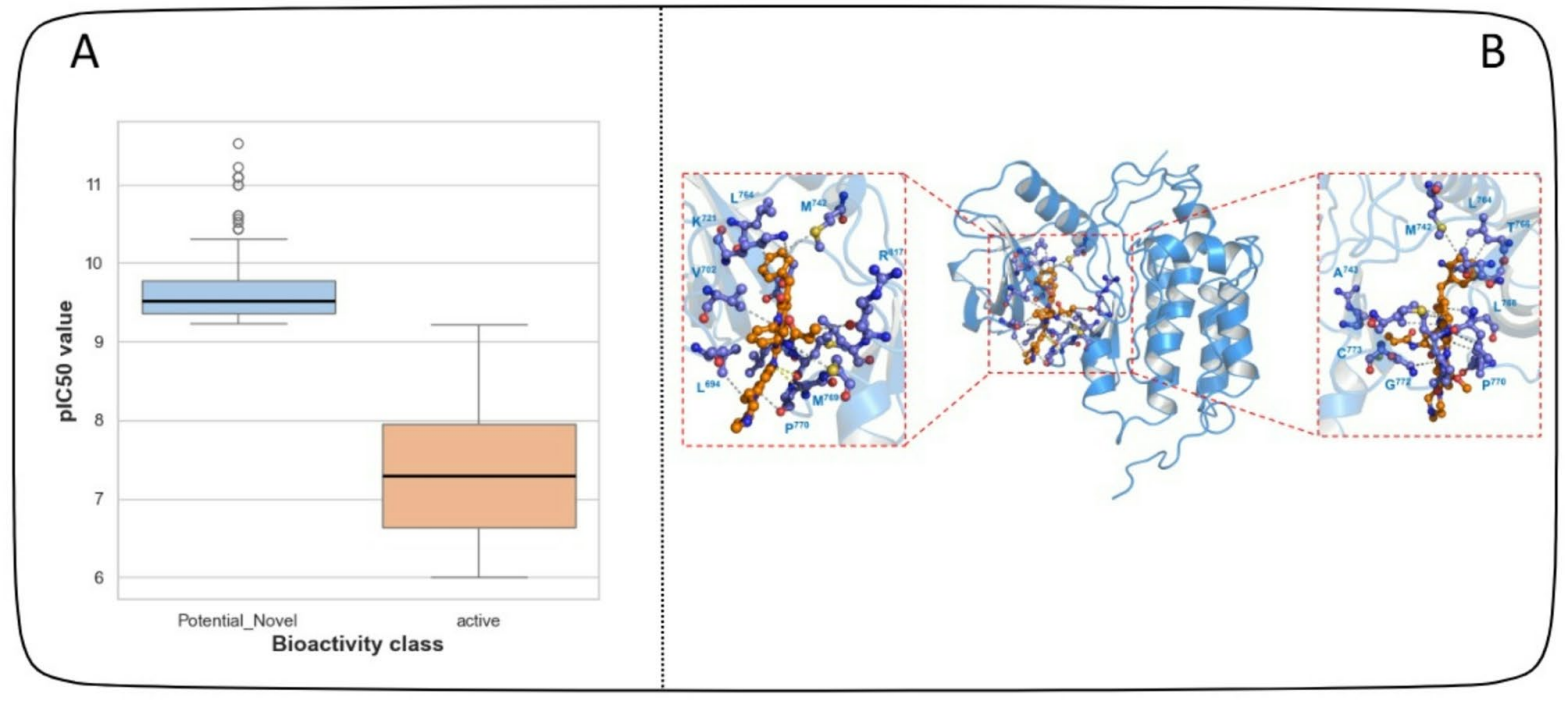
(**A**) pIC50 of potential inhibitors as compared with active inhibitors (**B**) Molecular Docking of Potential novel inhibitor with wild type EGFR. Hydrophobic interactions are shown in dotted line while hydrogen bonds and pi-pi interaction (green) are shown as solid line.

The predictive capability of DeepEGFR enabled the identification of 300 putative EGFR compounds, demonstrating strong potential for targeted cancer therapy. These underexplored compounds were selected based on their high confidence scores, structural similarity to known EGFR inhibitors, and distinct molecular features indicative of strong receptor binding. The finding of these inhibitors is very important as they expand the pool of potential drug candidates in cancers such as non-small cell lung cancer (NSCLC), and colorectal cancer, where EGFR plays a pivotal role in disease progression. Targeted cancer therapy has significantly improved treatment outcomes, and many patients develop resistance^57,58^. The putative inhibitors identified by DeepEGFR might exhibit diverse structural scaffolds. Additionally, these inhibitors may possess improved binding efficiency, selectivity, and reduced toxicity, making them attractive candidates for further drug development. The ability to predict previously unreported inhibitors highlights the strength of DeepEGFR in contributing to accelerating drug discovery and repurposing efforts. To validate the computational predictions, we analyzed these compounds’ half-maximal inhibitory concentration (IC50) values to further assess their potential. The IC50 values of the selected inhibitors were above the average IC50 of known active compounds (as shown in Fig. 8A, indicating their ability to effectively inhibit EGFR activity at therapeutically relevant concentrations. Higher IC50 values for active inhibitors suggest their strong binding affinity and high potency, reinforcing their role as promising candidates for further study. Next, molecular docking simulations were performed to assess these inhibitors’ binding affinity and stability at the EGFR active site. The docking results provided crucial insights into how these previously unreported compounds interact with EGFR’s key residues. Figure 8B illustrates the docking conformations of most promising inhibitors, showcasing their binding poses, and interaction patterns with the tyrosine kinase domain of EGFR. The analysis confirmed that these inhibitors engage in essential molecular interactions, such as hydrogen bonding, stacking, and hydrophobic interactions, which are critical for strong and selective EGFR inhibition. The docking results also revealed that some newly identified inhibitors displayed higher binding affinity than certain FDA-approved EGFR inhibitors, further supporting their therapeutic potential. However, while docking simulations provide valuable structural insights, additional computational and experimental validation is necessary to confirm their efficacy. To investigate further, we evaluated the ability to bind at the active site of EGFR, which is a critical aspect of their mechanism of action. Our analysis revealed that these inhibitors interact with key functional residues essential for EGFR activity, effectively competing with ATP for binding. This interaction is essential for blocking EGFR phosphorylation, inhibiting downstream oncogenic signalling. Given the central role of EGFR in tumor proliferation and survival, preventing its phosphorylation could significantly suppress cancer progression, making these novel inhibitors promising candidates for future therapeutic applications.

## Conclusion

In this study, we introduced DeepEGFR, a novel multi-class graph neural network method to accurately classify the bioactivity of EGFR inhibitors by integrating diverse molecular representations, including SMILES string and molecular fingerprints. DeepEGFR demonstrated exceptional performance, achieving approximately 94% F1-score across training and testing datasets, significantly outperforming traditional machine learning models such as Support Vector Machines (SVM), Random Forest, and Artificial Neural Networks (ANN). This highlights the strength of deep learning in handling complex molecular data and its potential for revolutionizing early-stage drug discovery. One of the key strengths of DeepEGFR lies in its ability to uncover mechanistic insights through feature importance analysis by identifying the top 20 most influential features, many of which align with the pharmacophoric elements of FDA-approved inhibition such as Afatinib, Gefitinib, Osimertinib, Dacombitinib and Erlotinib. Furthermore, the important features identified by DeepEGFR were used to select the most potent inhibitors from the dataset along with their IC50 values. expanding the landscape of potential EGFR inhibitors for EGFR-driven cancers, including non-small cell lung cancer (NSCLC), based on the validation and confirmation scores obtained from molecular docking simulations and molecular (MD) analysis. These underexplored potent compounds exhibit strong binding affinities and stable interactions with key residues in the EGFR active site. A key outcome of this study was the identification of 300 underexplored potent anti-EGFR compounds, which were further analyzed using molecular docking simulations and molecular dynamics (MD) analyses. These computational validation techniques confirmed that many of these inhibitors exhibited strong binding affinities and stable interactions with critical residues in the EGFR active site, suggesting their potential as effective EGFR-targeting agents. Furthermore, IC50 analysis indicated that several newly predicted compounds demonstrated activity levels compared to or exceeding those of known active compounds. It is worth to mention that our molecular dynamics simulations and MM/GBSA binding free energy analyses revealed that Top2 consistently exhibited stronger binding affinities than the clinically approved inhibitors, even in the presence of resistance-associated EGFR mutations (T790M, L858R, and C797S). Notably, Top2 maintained favorable energetic profiles across all EGFR variants, suggesting its potential as a robust inhibitor against both wild-type and mutant forms. These findings highlight Top2 as a promising candidate for overcoming resistance in EGFR-targeted therapies, as supported by the ΔTOTAL values presented in the MM/GBSA binding energy data as shown in Table S4.

Overall, these findings emphasise the transformative potential of deep learning in early-stage drug discovery and underscore the significance of experimental validation in advancing these compounds towards clinical applications. The underexplored potent anti-EGFR compounds identified in this study offer a robust foundation for future research, potentially leading to the development of more effective and selective EGFR-targeted therapies.

**Table 1:** PaDEL-descriptor derived fingerprint sets.

Fingerprint	Number	Description
2D atom pairs	780	Presence of atom pairs at various topological distances
2D atom pairs count	780	Count of atom pairs at various topological distances
CDK	1024	A molecular descriptor with 1024 data points, used for similarity searches with a depth of 8
CDK extended	1024	Extends the Fingerprinter with additional bits describing ring features
CDK graph only	1024	Specialized version of the Fingerprinter which does not take bond orders into account
Estate	79	Electrotopological state fragments
Klekota-Roth	4860	Presence of chemical substructures
Klekota-Roth count	4860	Count of chemical substructures
MACCS	166	Molecular ACCess System keys
PubChem	881	Pubchem fingerprint
Substructure	307	Presence of SMARTS Patterns for Functional Group Classification by Christian Laggner
Substructure count	307	Count of SMARTS Patterns for Functional Group Classification by Christian Laggner

**Table 2:** Summary of the top Kleokota-Roth features from the EGFR model along with their corresponding SMARTS patterns and description.

Rank	Features	SMARTS pattern	2D
1	KRFP1463	[!#1]C(= O)c1[cH][cH]c([OH])[cH][cH]1	4-Vinylphenol
2	KRFP25	[!#1][CH]([!#1])C(= O)[OH]	1,1-Ethanediol
3	KRFP1828	[!#1]c1[cH][cH]c2[cH][cH][cH][cH]c2n1	Quinoline
4	KRFP3459	CC1(C)CC(= CC(= O)C1)O	5,5-Dimethyl-cyclohex-2-enone-3-ol
5	KRFP1790	[!#1]c1[cH][cH]c(Cl)[cH]c1Br	1-Bromo-3-chlorobenzene
6	KRFP834	[!#1]c1[cH][cH]c2[cH]c([!#1])[cH][cH]c2[cH]1	Naphthalene
7	KRFP2025	[!#1]c1[cH]nc([!#1])n[cH]1	Pyrimidine
8	KRFP1462	[!#1]C(= O)c1[cH][cH]c([CH3])c([cH]1)S(= O)(= O)[!#1]	5-Acetyl-2-methylbenzenesulfinate
9	KRFP1576	[!#1]c1[cH][cH][cH][cH]c1C(= O)c2[cH]c([!#1])nnc2[!#1]	Phenyl(pyridazin-4-yl)methanone
11	KRFP4820	OCCc1ccccc1	Phenylethyl alcohol
12	KRFP1894	[!#1]c1[cH][cH]c2O[CH2][CH2]Oc2[cH]1	1,4-Benzodioxan
13	KRFP4522	O = CCCCC = O	Glutaral
14	KRFP3045	C1c2ccccc2Oc3ccccc13	Anthracene
15	KRFP4412	O = C1CCCN1c2ccccc2	1-Benzyl-2-pyrrolidinone

## Supplementary Information

Below is the link to the electronic supplementary material.

Supplementary Material 1

## Data Availability

The data and code are available. https://github.com/CATG-Github/DeepEGFR-A-Graph-Neural-Network-Method-for-Accurate-Classification-of-Bioactivity-of-EGFR-Inhibitors.
