# Supplementary material for: DeepEGFR a graph neural network for bioactivity classification of EGFR inhibitors
Source: Sci Rep. 2025 Oct 31;15:38236. doi: 10.1038/s41598-025-22126-8 (PMC12578785; doi:10.1038/s41598-025-22126-8)
Supplement: Supplementary file 1 — Supplementary Material 1 [file 41598_2025_22126_MOESM1_ESM.pdf]

# Supplementary file

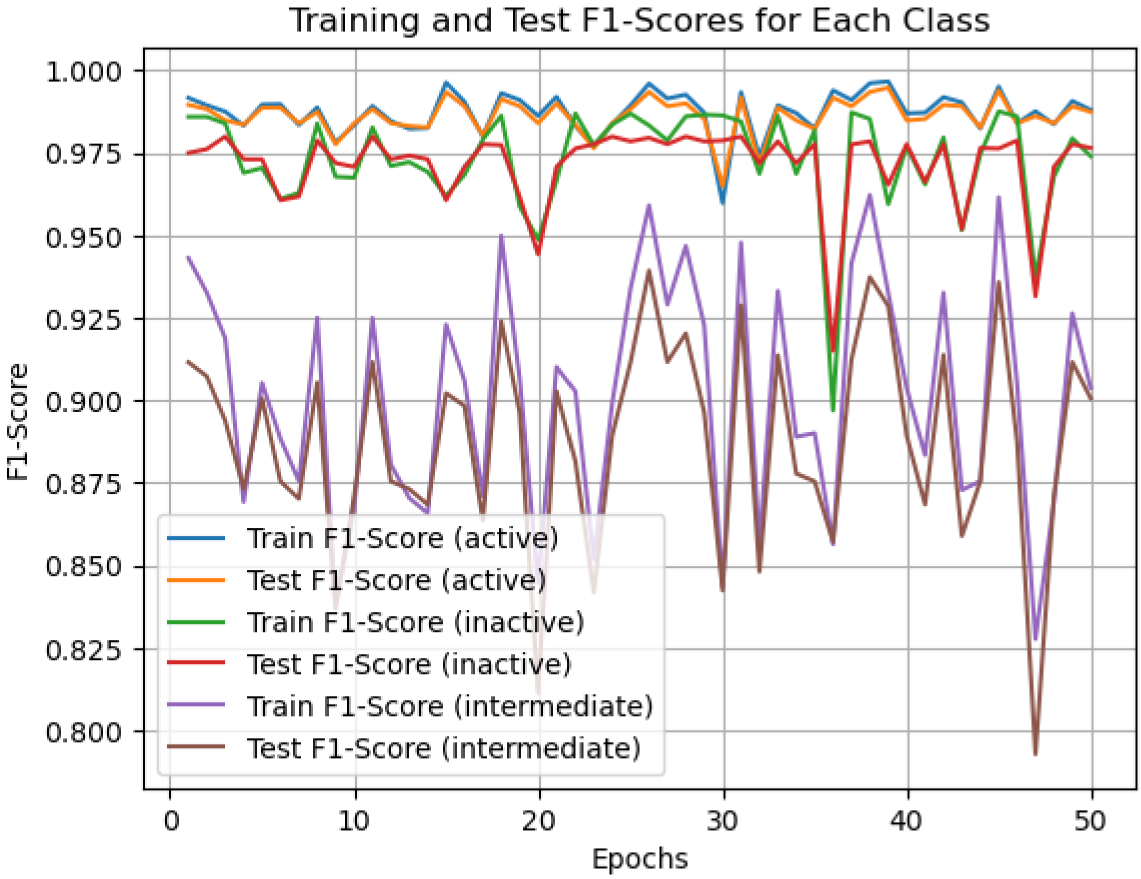


**Figure S1:** Training and testing for each activity class (Active, Inactive, Intermediate). It demonstrates consistent performance across the three classes, with minimal fluctuations in both training and testing phases. The model achieves high F1-scores, nearing 0.94 for all classes, indicating strong generalization and balanced classification performance.

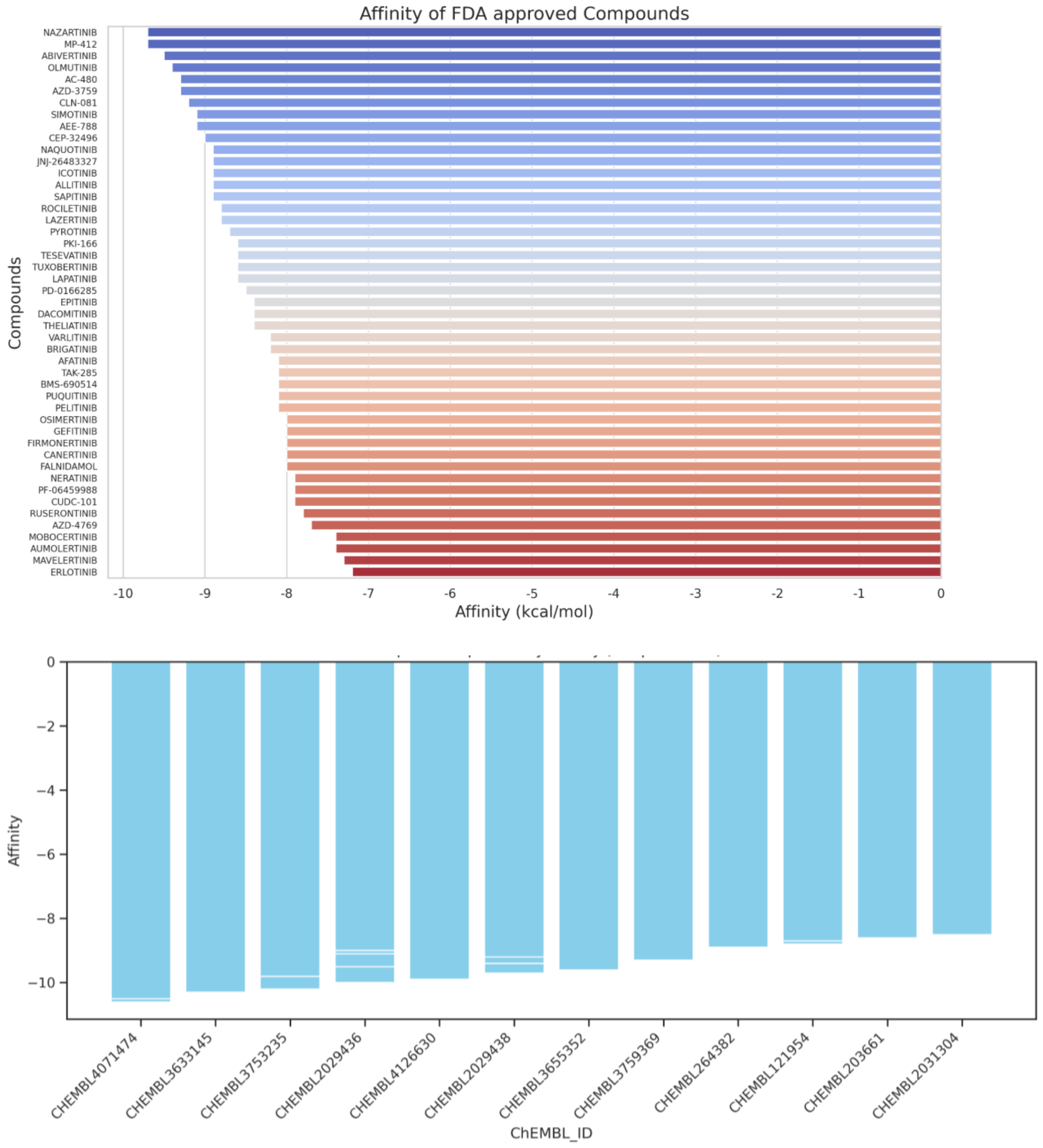


**Figure S2:** (A)Affinity (kcal/mo) derived from molecular docking of FDA-approved drugs and top 12 compounds with high affinity from the list of 300 compounds

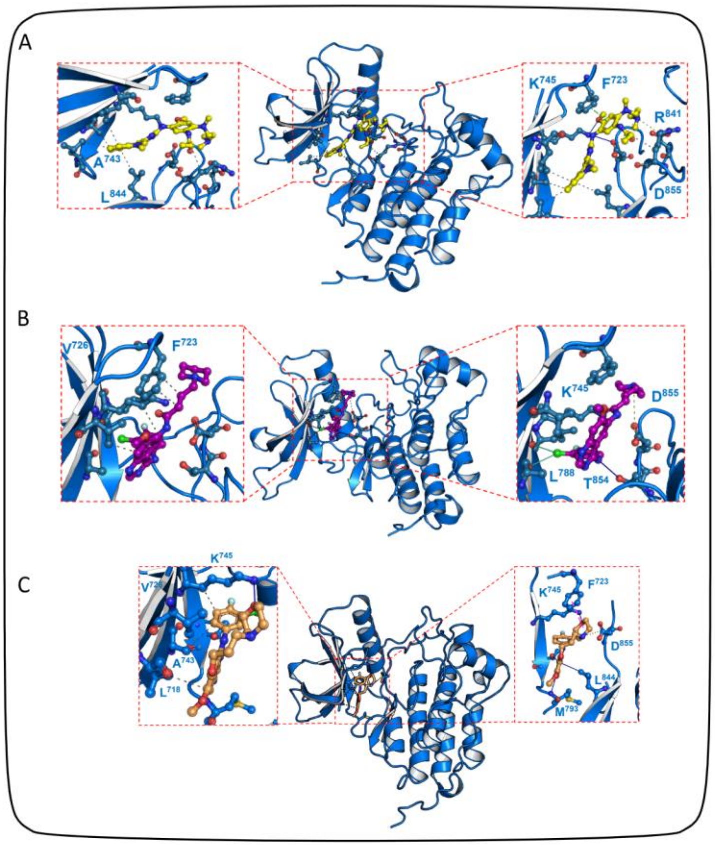


**Figure S3**: Molecular docking of FDA-approved drugs (A) Gefitinib (B) Osimertinib and (C) Decomtinib. The interactions are shown by dotted line between residues and ligands, pi-pi interactions are shown in orange dotted line while as hydrogen bonds and hydrophobic bonds are shown in solid and dotted grey lines, respectively.

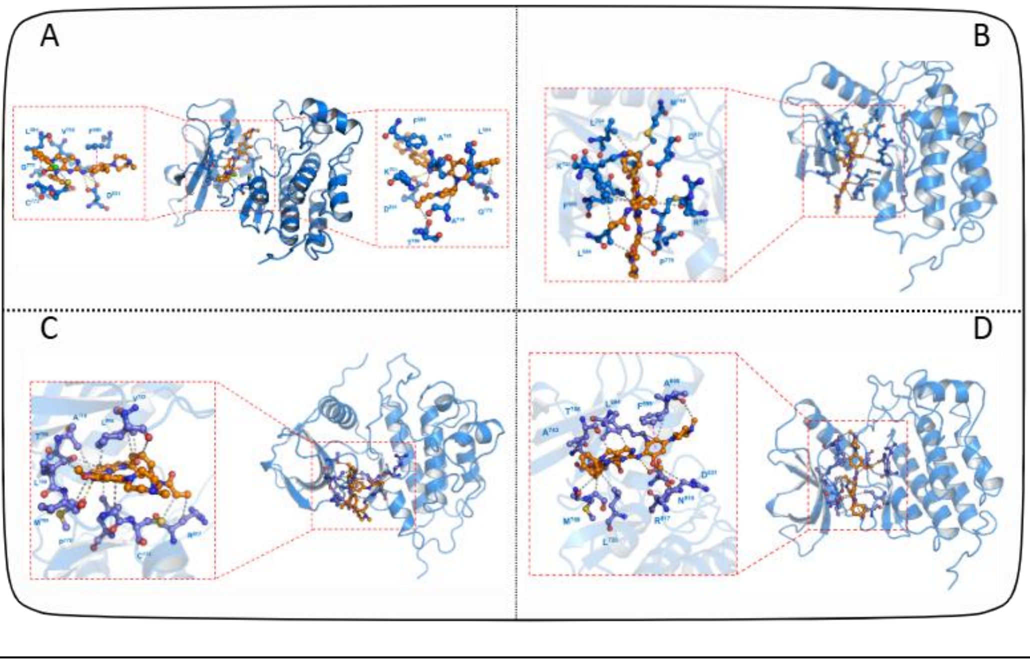


**Figure S4:** Docking result of top novel potential compound with EGFR.The interactions are shown by dotted line between residues and ligands, pi-pi interactions are shown in orange dotted line while as hydrogen bonds and hydrophobic bonds are shown in solid and dotted grey lines, respectively.

**Table S1:** Summary of the top PubChem features from the EGFR model along with their corresponding SMARTS patterns and description

| Rank | Features | SMARTS pattern | 2D |
|---|---|---|---|
| **1** | PubChemFP772 | Nc1c(Br)cccc1 | 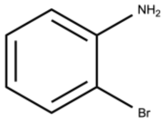 2-bromoaniline |
| **2** | PubChemFP861 | CC1C(O)CCC1 | 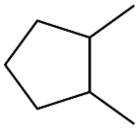 1,2-Dimethylcyclopentane |
| **3** | PubChemFP568 | N#C-C-C | 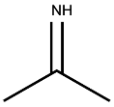 2-Propanimine |
| **4** | PubChemFP665 | N-C:C-C-C | 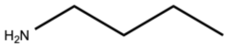 N-butylamine |
| **5** | PubChemFP189 | >= 2 unsaturated non-aromatic carbon-only ring size 6 | |
| **6** | PubChemFP672 | O=C-C=C-[#1] | 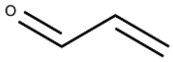 Acrolein |
| **7** | PubChemFP661 | O-C-O-C-C | 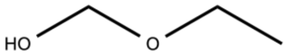 Ethoxymethanol |
| **8** | PubChemFP728 | Nc1ccc(N)cc1 | 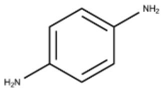 p-Phenylenediamine |

**Table S2:** Ablation studies were conducted to quantify the contribution of molecular fingerprints to the overall performance of the DeepEGFR model. It represents the performance of the complete DeepEGFR model using both SMILES-derived graph structures and molecular fingerprints (Klekota-Roth and PubChem). The absence of fingerprints led to a consistent decrease in classification performance

| | | **DeepEGFR (SMILES and Klekota-Roth)** | **DeepEGFR (SMILES only)** | **DeepEGFR (Klekota-Roth only)** |
|---|---|---|---|---|
| **Epoch** | **Class** | **F1-Score** | **F1-Score** | **F1-Score** |
| 1 | active | 0.993101938 | 0.794982 | 0.803579 |
| 1 | inactive | 0.95840868 | 0.818029 | 0.756769 |
| 1 | intermediate | 0.904694168 | 0.80928 | 0.750143 |
| 2 | active | 0.986407767 | 0.803946 | 0.774262 |
| 2 | inactive | 0.818114328 | 0.786241 | 0.808996 |

| | | | | |
|---|---|---|---|---|
| 2 | intermediate | 0.762711864 | 0.78624 | 0.756944 |
| 3 | active | 0.991434457 | 0.782323 | 0.787049 |
| 3 | inactive | 0.979823455 | 0.814647 | 0.793313 |
| 3 | intermediate | 0.933949192 | 0.804045 | 0.756635 |
| 4 | active | 0.984496124 | 0.808323 | 0.790975 |
| 4 | inactive | 0.975429975 | 0.780823 | 0.765745 |
| 4 | intermediate | 0.888888889 | 0.818796 | 0.784261 |
| 5 | active | 0.990895296 | 0.813298 | 0.784347 |
| 5 | inactive | 0.95670475 | 0.788494 | 0.777504 |
| 5 | intermediate | 0.886454183 | 0.787273 | 0.74632 |
| 6 | active | 0.986940097 | 0.787336 | 0.798471 |
| 6 | inactive | 0.974293875 | 0.79217 | 0.762455 |
| 6 | intermediate | 0.905783582 | 0.80099 | 0.753056 |
| 7 | active | 0.992724509 | 0.797278 | 0.742854 |
| 7 | inactive | 0.975429975 | 0.791649 | 0.781363 |
| 7 | intermediate | 0.929832936 | 0.804474 | 0.78743 |
| 8 | active | 0.978272981 | 0.78558 | 0.741161 |
| 8 | inactive | 0.906552707 | 0.791686 | 0.775847 |
| 8 | intermediate | 0.747951807 | 0.794654 | 0.755855 |
| 9 | active | 0.98715319 | 0.798243 | 0.785162 |
| 9 | inactive | 0.806451613 | 0.811407 | 0.752206 |
| 9 | intermediate | 0.758646902 | 0.787987 | 0.788366 |
| 10 | active | 0.993867718 | 0.800569 | 0.767071 |
| 10 | inactive | 0.935057302 | 0.803697 | 0.805571 |
| 10 | intermediate | 0.863209877 | 0.781858 | 0.749626 |
| 11 | active | 0.985663469 | 0.804302 | 0.763875 |
| 11 | inactive | 0.97631498 | 0.786821 | 0.747943 |
| 11 | intermediate | 0.896653543 | 0.782602 | 0.804729 |
| 12 | active | 0.98122519 | 0.817955 | 0.801414 |
| 12 | inactive | 0.855192239 | 0.818625 | 0.758056 |
| 12 | intermediate | 0.76465798 | 0.812336 | 0.786199 |
| 13 | active | 0.984602132 | 0.792185 | 0.797206 |
| 13 | inactive | 0.975970425 | 0.783907 | 0.778864 |
| 13 | intermediate | 0.89097188 | 0.807369 | 0.777076 |
| 14 | active | 0.992827646 | 0.797606 | 0.75693 |
| 14 | inactive | 0.958433735 | 0.784882 | 0.746517 |
| 14 | intermediate | 0.899901865 | 0.799807 | 0.802805 |
| 15 | active | 0.986283186 | 0.781376 | 0.803029 |
| 15 | inactive | 0.915683453 | 0.816373 | 0.784317 |
| 15 | intermediate | 0.79608802 | 0.790351 | 0.763732 |
| 16 | active | 0.984939092 | 0.806501 | 0.764445 |
| 16 | inactive | 0.920451258 | 0.792468 | 0.790817 |
| 16 | intermediate | 0.802701399 | 0.800803 | 0.802798 |
| 17 | active | 0.99410609 | 0.801868 | 0.802096 |
| 17 | inactive | 0.956364731 | 0.787394 | 0.794591 |
| 17 | intermediate | 0.904096386 | 0.818783 | 0.784942 |
| 18 | active | 0.989927434 | 0.811005 | 0.74589 |
| 18 | inactive | 0.968017058 | 0.81758 | 0.751314 |

| | | | | |
|---|---|---|---|---|
| 18 | intermediate | 0.903131115 | 0.815793 | 0.802899 |
| 19 | active | 0.980068581 | 0.803916 | 0.78245 |
| 19 | inactive | 0.934311437 | 0.816875 | 0.740644 |
| 19 | intermediate | 0.828174069 | 0.78354 | 0.747103 |
| 20 | active | 0.990682557 | 0.787839 | 0.786445 |
| 20 | inactive | 0.979069041 | 0.781809 | 0.740354 |
| 20 | intermediate | 0.928135275 | 0.793013 | 0.751257 |
| 21 | active | 0.994650071 | 0.795547 | 0.778411 |
| 21 | inactive | 0.956338452 | 0.790854 | 0.788433 |
| 21 | intermediate | 0.906730769 | 0.81315 | 0.785637 |
| 22 | active | 0.96103601 | 0.79427 | 0.755699 |
| 22 | inactive | 0.942256441 | 0.791237 | 0.789853 |
| 22 | intermediate | 0.77394958 | 0.801708 | 0.756607 |
| 23 | active | 0.981330472 | 0.785637 | 0.762778 |
| 23 | inactive | 0.960980329 | 0.812088 | 0.792254 |
| 23 | intermediate | 0.862085086 | 0.782982 | 0.785474 |
| 24 | active | 0.994019789 | 0.819475 | 0.799446 |
| 24 | inactive | 0.954668268 | 0.81089 | 0.786033 |
| 24 | intermediate | 0.898622047 | 0.787949 | 0.779782 |
| 25 | active | 0.988970588 | 0.780221 | 0.746557 |
| 25 | inactive | 0.923076923 | 0.812618 | 0.76574 |
| 25 | intermediate | 0.859454855 | 0.808274 | 0.758564 |
| 26 | active | 0.982068077 | 0.80916 | 0.757079 |
| 26 | inactive | 0.75703125 | 0.810851 | 0.808111 |
| 26 | intermediate | 0.706363975 | 0.782962 | 0.767517 |
| 27 | active | 0.980593974 | 0.794339 | 0.802443 |
| 27 | inactive | 0.954574951 | 0.784635 | 0.78418 |
| 27 | intermediate | 0.850767085 | 0.814524 | 0.795637 |
| 28 | active | 0.987790384 | 0.804932 | 0.775185 |
| 28 | inactive | 0.977265649 | 0.793236 | 0.780383 |
| 28 | intermediate | 0.911174785 | 0.782542 | 0.774476 |
| 29 | active | 0.983546618 | 0.792439 | 0.753667 |
| 29 | inactive | 0.974683544 | 0.793007 | 0.790572 |
| 29 | intermediate | 0.889100428 | 0.809184 | 0.759654 |
| 30 | active | 0.987262522 | 0.805502 | 0.741702 |
| 30 | inactive | 0.957254666 | 0.815489 | 0.785183 |
| 30 | intermediate | 0.868287741 | 0.798889 | 0.752398 |
| 31 | active | 0.991219512 | 0.784784 | 0.805832 |
| 31 | inactive | 0.897692043 | 0.80853 | 0.806775 |
| 31 | intermediate | 0.844572368 | 0.810431 | 0.804041 |
| 32 | active | 0.993655655 | 0.802451 | 0.765911 |
| 32 | inactive | 0.954381753 | 0.810839 | 0.741082 |
| 32 | intermediate | 0.899328859 | 0.799752 | 0.804982 |
| 33 | active | 0.988649876 | 0.800909 | 0.769973 |
| 33 | inactive | 0.862759114 | 0.797102 | 0.807666 |
| 33 | intermediate | 0.80525687 | 0.781017 | 0.807453 |
| 34 | active | 0.984280792 | 0.784316 | 0.799711 |
| 34 | inactive | 0.956390977 | 0.781257 | 0.760611 |

| | | | | |
|---|---|---|---|---|
| 34 | intermediate | 0.850539291 | 0.805456 | 0.766957 |
| 35 | active | 0.98960814 | 0.792574 | 0.79958 |
| 35 | inactive | 0.977160494 | 0.800343 | 0.762185 |
| 35 | intermediate | 0.918347743 | 0.816303 | 0.751864 |
| 36 | active | 0.992402865 | 0.789972 | 0.778976 |
| 36 | inactive | 0.891440501 | 0.796415 | 0.805531 |
| 36 | intermediate | 0.845469256 | 0.810222 | 0.788722 |
| 37 | active | 0.988431182 | 0.789152 | 0.779904 |
| 37 | inactive | 0.976572133 | 0.783079 | 0.746802 |
| 37 | intermediate | 0.911465893 | 0.79159 | 0.783051 |
| 38 | active | 0.988006483 | 0.786449 | 0.809304 |
| 38 | inactive | 0.969493594 | 0.817188 | 0.749806 |
| 38 | intermediate | 0.895905279 | 0.812325 | 0.776283 |
| 39 | active | 0.994878501 | 0.805336 | 0.801416 |
| 39 | inactive | 0.974753695 | 0.814858 | 0.791854 |
| 39 | intermediate | 0.939578454 | 0.812147 | 0.788791 |
| 40 | active | 0.989503301 | 0.787463 | 0.789174 |
| 40 | inactive | 0.948717949 | 0.815702 | 0.765164 |
| 40 | intermediate | 0.863104326 | 0.801574 | 0.760551 |
| 41 | active | 0.993474005 | 0.812298 | 0.796655 |
| 41 | inactive | 0.963906582 | 0.815844 | 0.796708 |
| 41 | intermediate | 0.913484775 | 0.79272 | 0.800695 |
| 42 | active | 0.993478261 | 0.784402 | 0.803927 |
| 42 | inactive | 0.949253731 | 0.789117 | 0.775794 |
| 42 | intermediate | 0.885572139 | 0.797084 | 0.775106 |
| 43 | active | 0.993776613 | 0.812721 | 0.795881 |
| 43 | inactive | 0.974992282 | 0.814429 | 0.785497 |
| 43 | intermediate | 0.936170213 | 0.780278 | 0.789138 |
| 44 | active | 0.984287559 | 0.80043 | 0.795705 |
| 44 | inactive | 0.976256553 | 0.796696 | 0.8023 |
| 44 | intermediate | 0.889876543 | 0.788884 | 0.76366 |
| 45 | active | 0.992829205 | 0.784795 | 0.766291 |
| 45 | inactive | 0.864884135 | 0.793505 | 0.746579 |
| 45 | intermediate | 0.825558604 | 0.817716 | 0.78048 |
| 46 | active | 0.991859329 | 0.792928 | 0.742516 |
| 46 | inactive | 0.9566787 | 0.800752 | 0.772592 |
| 46 | intermediate | 0.891744933 | 0.808121 | 0.777985 |
| 47 | active | 0.991541965 | 0.794545 | 0.760058 |
| 47 | inactive | 0.951822917 | 0.818871 | 0.781358 |
| 47 | intermediate | 0.900264784 | 0.818498 | 0.742135 |
| 48 | active | 0.987046632 | 0.790071 | 0.742614 |
| 48 | inactive | 0.808685885 | 0.79989 | 0.797582 |
| 48 | intermediate | 0.759619048 | 0.792035 | 0.765213 |
| 49 | active | 0.990148317 | 0.791394 | 0.748894 |
| 49 | inactive | 0.973808772 | 0.781475 | 0.776557 |
| 49 | intermediate | 0.919220056 | 0.804383 | 0.7939 |
| 50 | active | 0.989865609 | 0.800107 | 0.755107 |
| 50 | inactive | 0.930409357 | 0.782059 | 0.783602 |

| 50 | intermediate | 0.839961203 | 0.791146 | 0.745974 |
|---|---|---|---|---|

**Table S3: S**ummary of Binding Free Energy Calculations for EGFR Inhibitors. In this table , the calculated binding free energies and their components for five FDA-approved EGFR inhibitors (Afatinib, Dacomitinib, Osimertinib, Gefitinib, and Erlotinib) and five underinvestigated potential inhibitors (Top1, Top2, Top3, Top4, and Top5) using the MM-PBSA method.

| Molecule Name | Type | ΔGtotal (kJ/mol) | ΔGele (kJ/mol) | ΔGvdw (kJ/mol) | ΔGpol (kJ/mol) | ΔGnonpol (kJ/mol) |
|---|---|---|---|---|---|---|
| Afatinib | FDA-Approved | -32.75 | -9.24 | -45 | 8.67 | -5.67 |
| Dacomitinib | FDA-Approved | -29.61 | -6.18 | -43.18 | 12.72 | -5.33 |
| Osimertinib | FDA-Approved | -11.97 | -5.27 | -28.67 | 14.75 | -3.32 |
| Erlotinib | FDA-Approved | -26.43 | -9.77 | -36.08 | 24.05 | -4.63 |
| Top1 | Underinvestigated | -25.08 | -15.81 | -41.62 | 47.37 | -5.02 |
| Top2 | Underinvestigated | -35.55 | -26.39 | -55.5 | 40.05 | -6.4 |
| Top3 | Underinvestigated | -20.99 | -0.34 | -38.79 | 22.85 | -4.72 |
| Top4 | Underinvestigated | -24.18 | -14.92 | -37.38 | 22.22 | -6.4 |
| Top5 | Underinvestigated | -13.75 | -9.41 | -30.84 | 11.06 | -3.38 |

ΔGtotal: Total binding free energy, representing the overall energy change upon ligand binding. ΔGele: Electrostatic contribution to the binding free energy, reflecting the charged interactions between the protein and the ligand. ΔGvdw: van der Waals contribution to the binding free energy, representing the attractive and repulsive forces arising from the temporary fluctuations in electron distribution. ΔGpol: Polar solvation energy, representing the energy change associated with the desolvation of polar and charged groups upon complex formation.ΔGnonpol: Non-polar solvation energy, representing the favorable energy change associated with the burial of non-polar surface area upon binding.

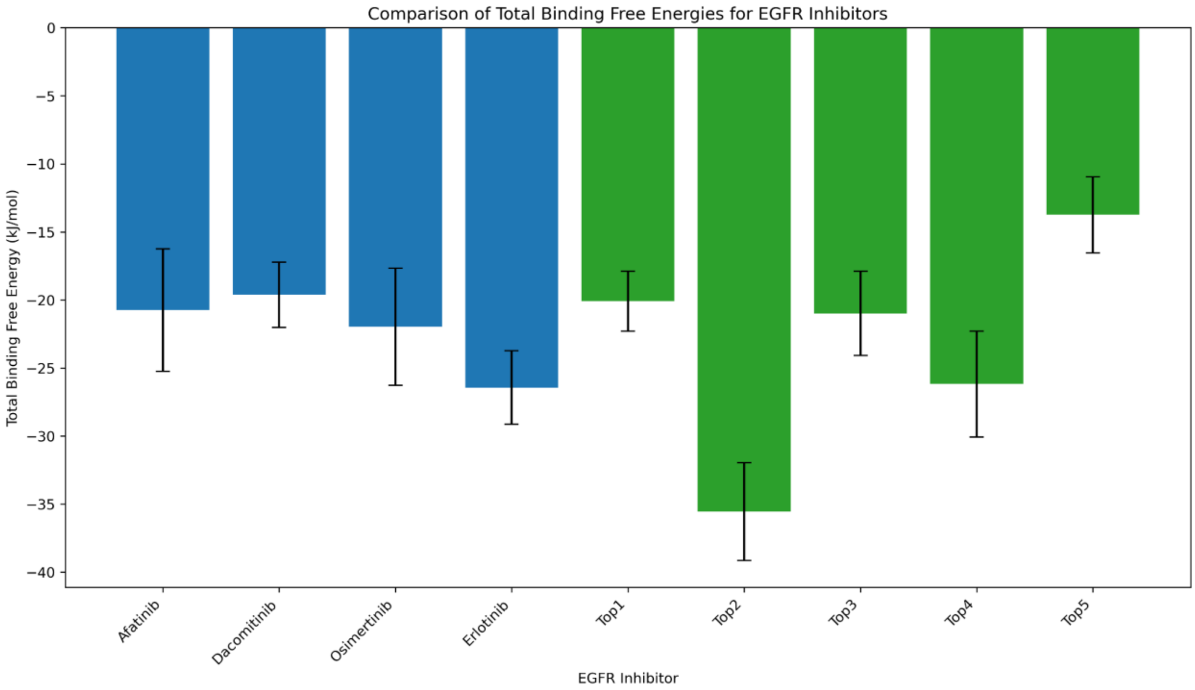


**Fig S4 Comparison of Total Binding Free Energies for EGFR Inhibitors.** Bar plot showing the calculated total binding free energies (ΔGtotal) for five FDA-approved EGFR inhibitors (Afatinib, Dacomitinib, Osimertinib, Gefitinib, Erlotinib) and five under investigated inhibitors (Top1, Top2, Top3, Top4, Top5). Error bars represent the standard error of the mean.

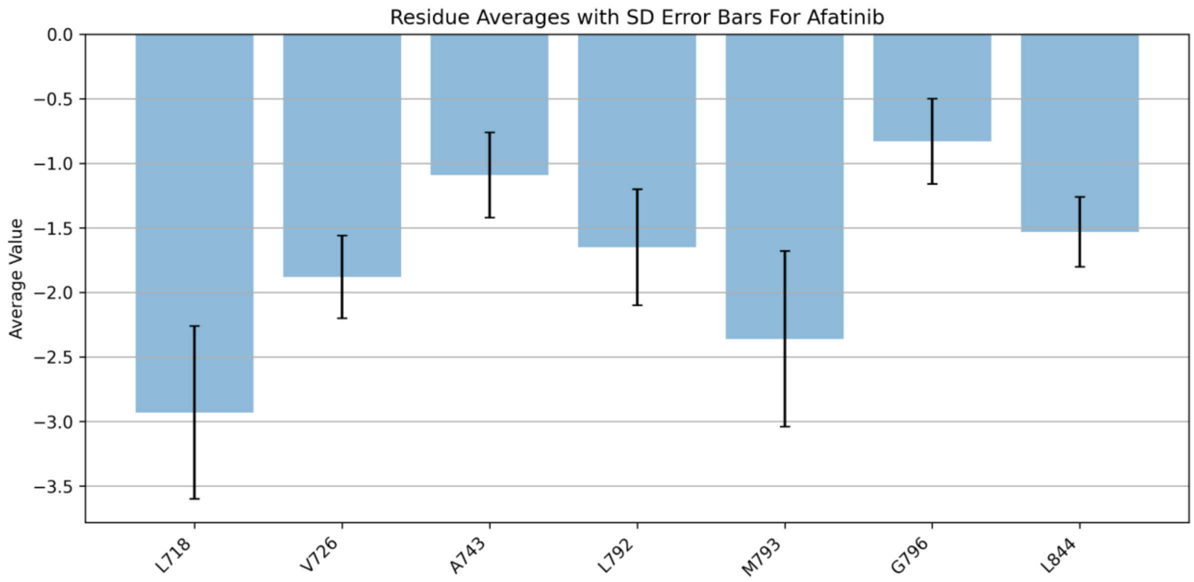
Residue Averages with SD Error Bars For Afatinib
Average Value
0.0
−0.5
−1.0
−1.5
−2.0
−2.5
−3.0
−3.5
L718
V726
A743
L792
M793
G796
L844

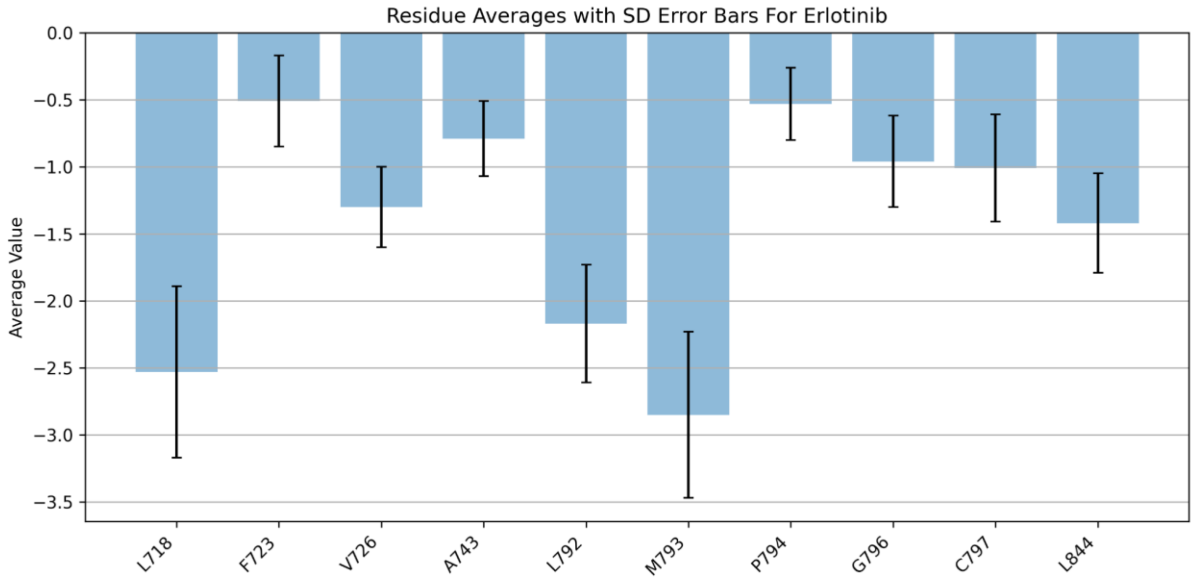
Residue Averages with SD Error Bars For Erlotinib
Average Value
0.0
−0.5
−1.0
−1.5
−2.0
−2.5
−3.0
−3.5
L718
F723
V726
A743
L792
M793
P794
G796
C797
L844

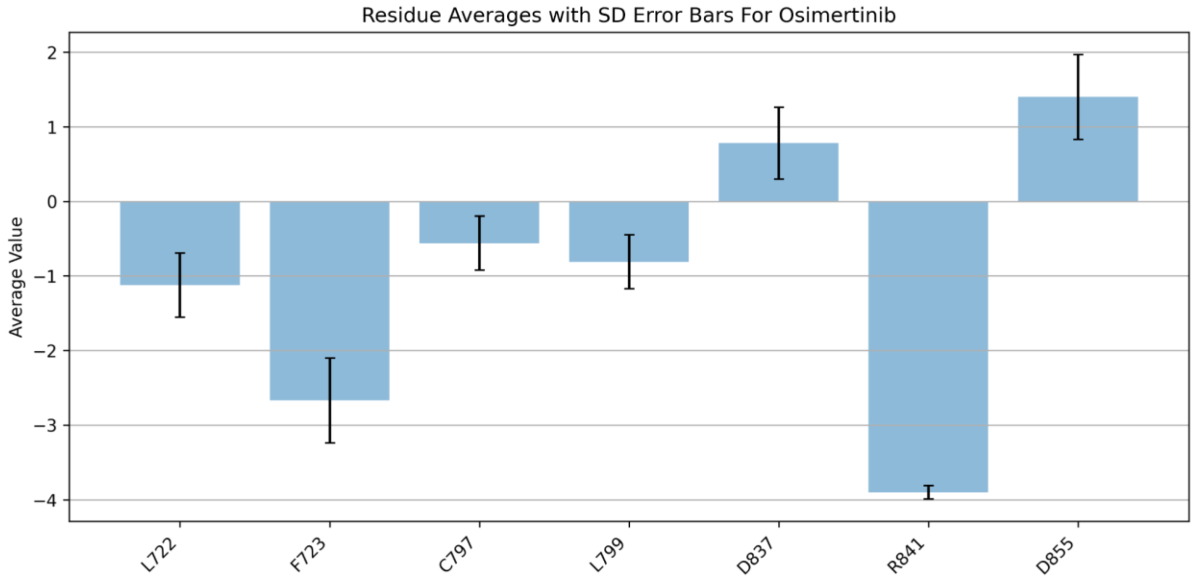
Residue Averages with SD Error Bars For Osimertinib
Average Value
2
1
0
−1
−2
−3
−4
L722
F723
C797
L799
D837
R841
D855

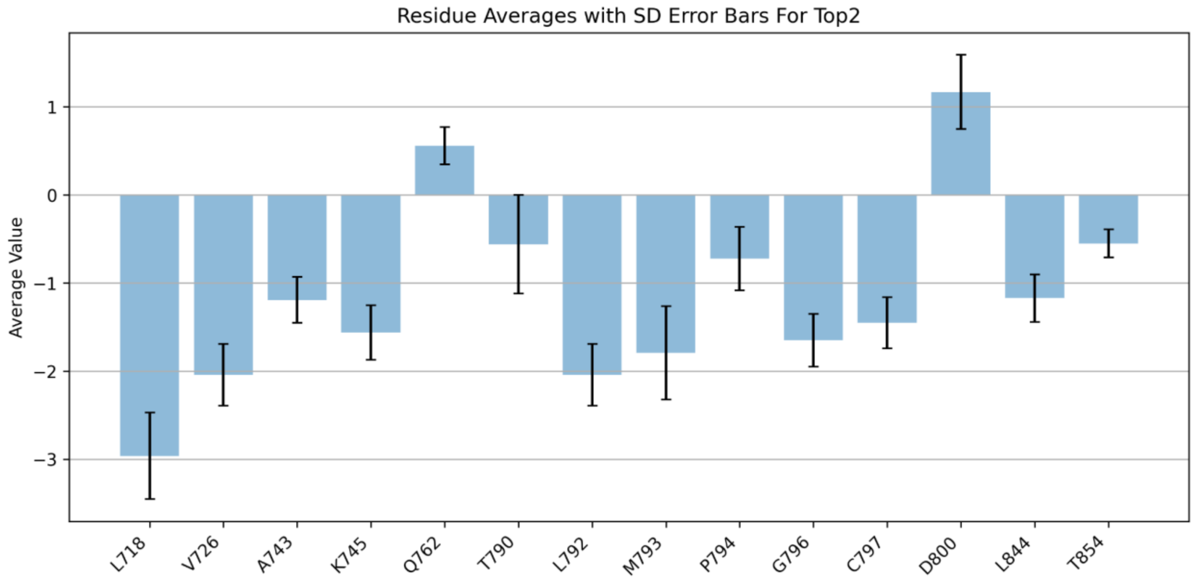


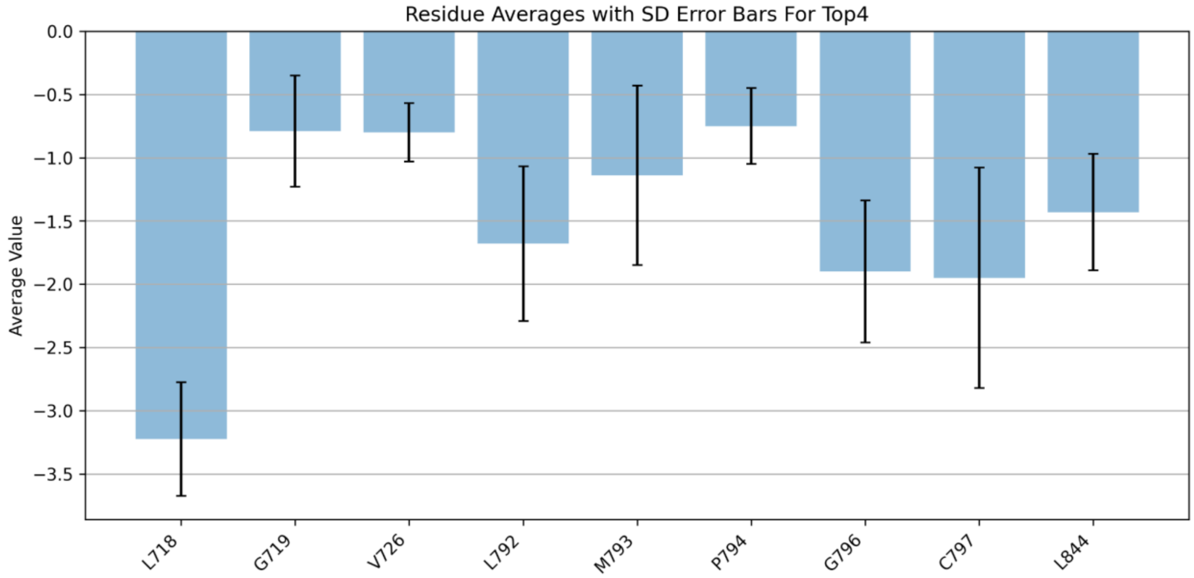


**Figure S5:** Per-residue energy decomposition analysis for (A) Afatinib, (B) Osimertinib, © Erlotinib, (D) Top2, and (E) Top4. The energy contribution (kcal/mol) of each amino acid residue in the binding site is plotted for each inhibitor. Residues with negative values contribute favorably to binding, while those with positive values are unfavorable. Key residues contributing significantly to the binding of each inhibitor are labeled

(**A**)

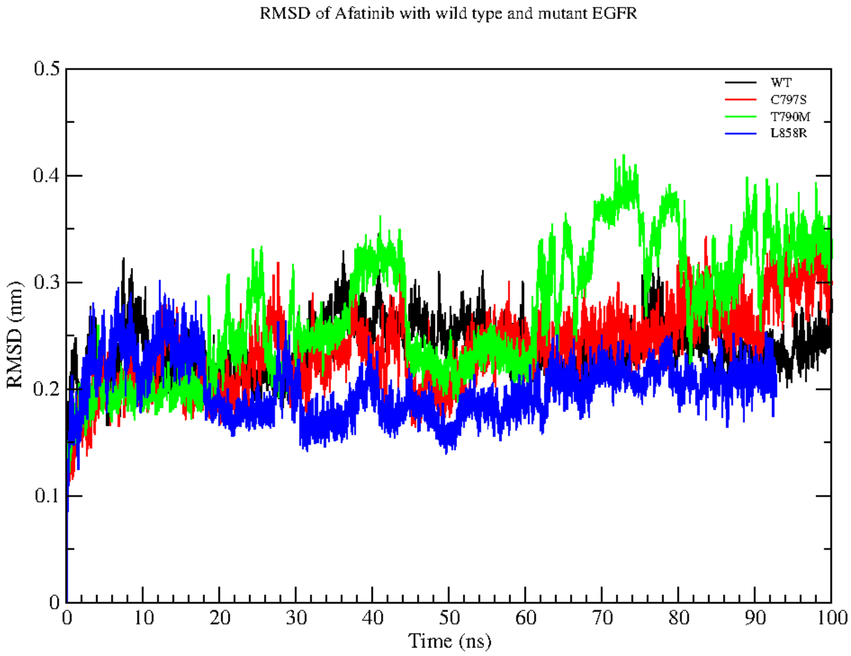
RMSD of Afatinib with wild type and mutant EGFR
WT
C797S
T790M
L858R
RMSD (nm)
0
0.1
0.2
0.3
0.4
0.5
0
10
20
30
40
50
60
70
80
90
100
Time (ns)

(**B**)

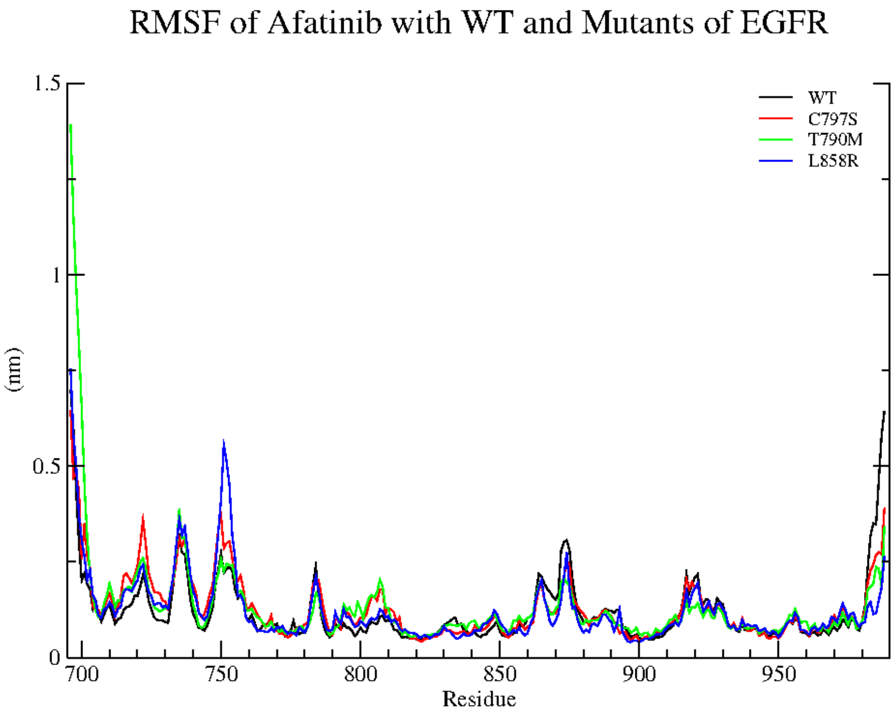
RMSF of Afatinib with WT and Mutants of EGFR
WT
C797S
T790M
L858R
(nm)
0
0.5
1
1.5
700
750
800
850
900
950
Residue

(**C**)

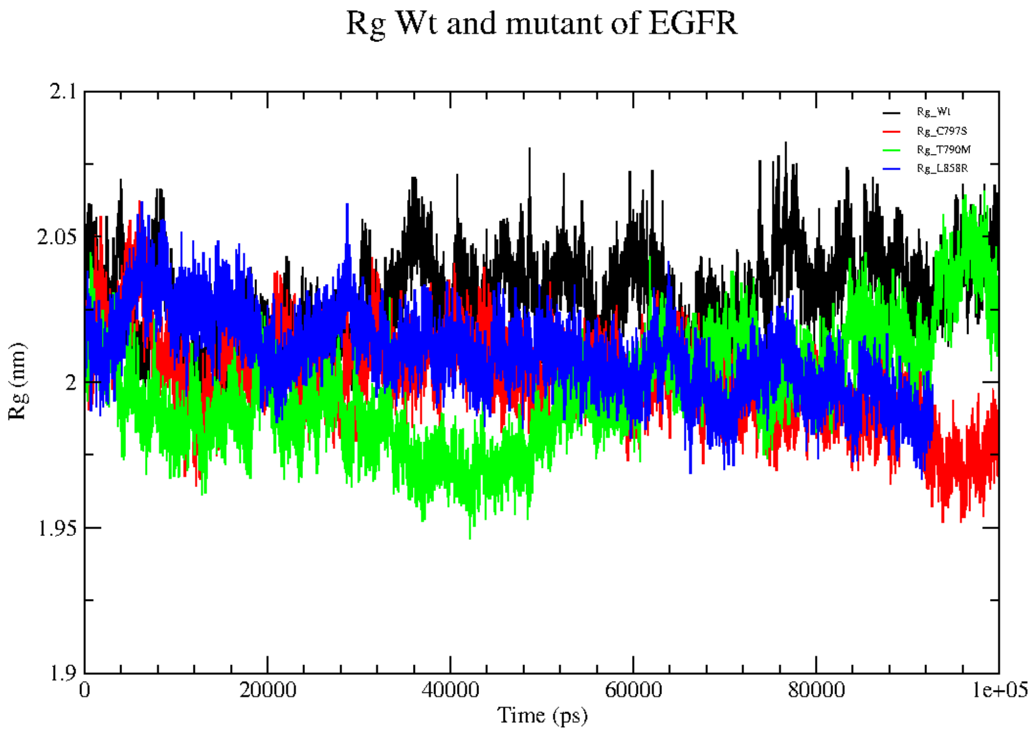


**Figure S6.** Comparative molecular dynamics analysis of EGFR wild-type and mutant (C797S, T790M, L858R) complexes with Afatinib over a 100 ns simulation. (**A**) Root Mean Square Deviation (RMSD), profiles illustrating the overall structural stability and equilibration of the EGFR wild-type and mutant protein backbones in complex with each inhibitor.(**B**) Root Mean Square Fluctuation (RMSF) analysis depicting residue-level flexibility across the EGFR variants, providing insight into local conformational dynamics upon ligand binding.(**C**) Radius of gyration (Rg) plots evaluating the compactness and structural integrity of each protein–ligand complex throughout the simulation.

(**A**)

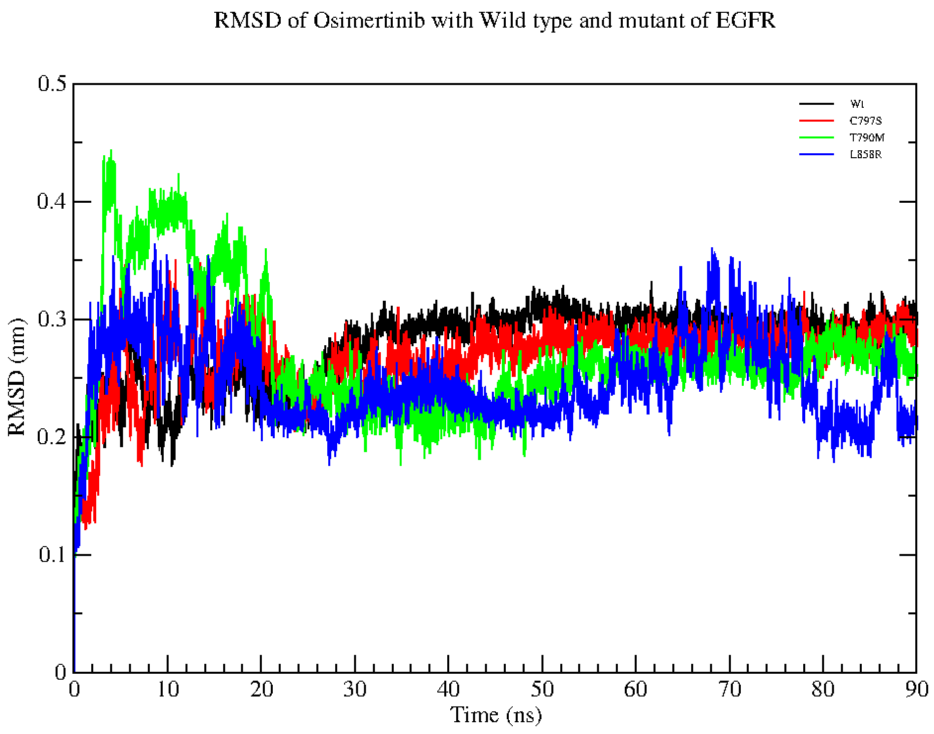

RMSD of Osimertinib with Wild type and mutant of EGFR
Wt
C797S
T790M
L858R
RMSD (nm)
Time (ns)
0
0.1
0.2
0.3
0.4
0.5
0
10
20
30
40
50
60
70
80
90


(B)

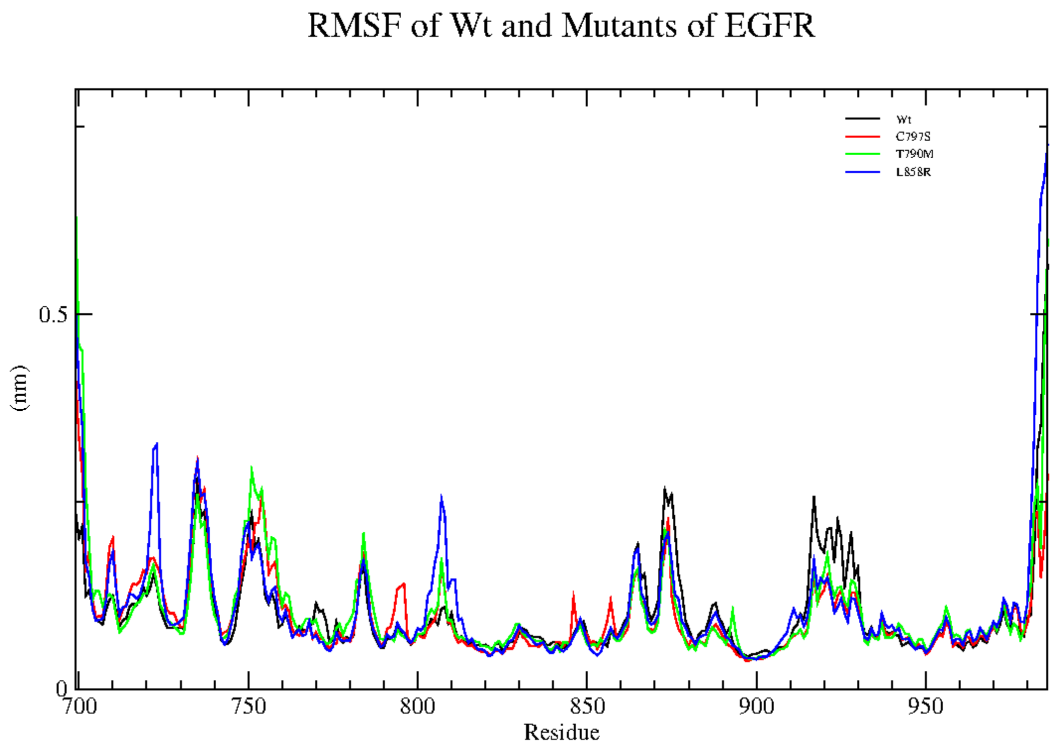

RMSF of Wt and Mutants of EGFR
Wt
C797S
T790M
L858R
(nm)
Residue
0
0.5
700
750
800
850
900
950


(C)

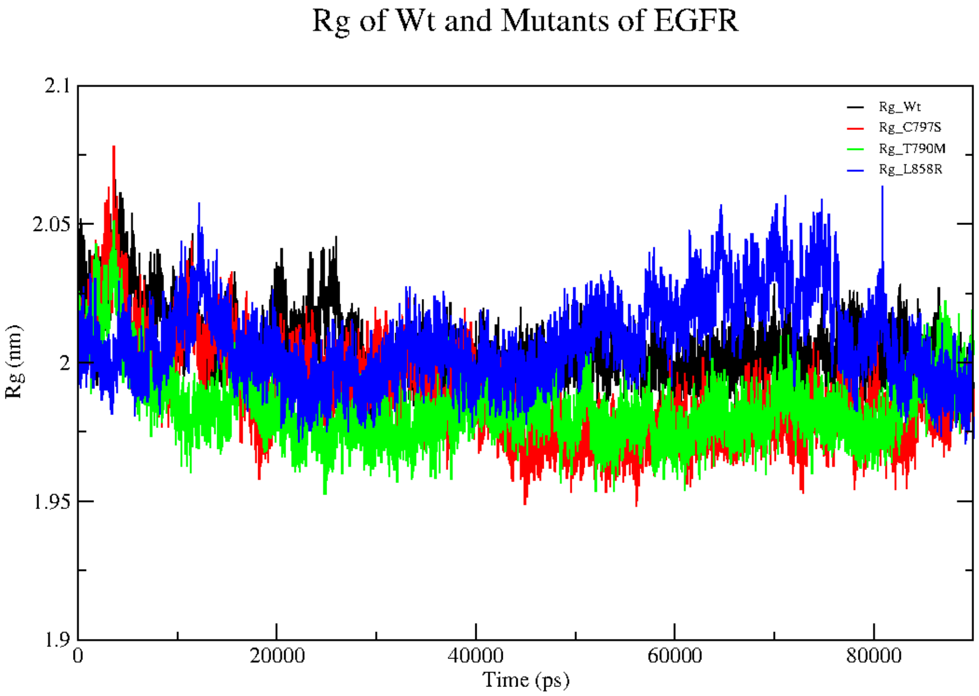


**Figure S7.** Comparative molecular dynamics analysis of EGFR wild-type and mutant complexes (C797S, T790M, L858R) with Osimertinib over a 100 ns simulation. (**A**) Root Mean Square Deviation (RMSD), profiles illustrating the overall structural stability and equilibration of the EGFR wild-type and mutant protein backbones in complex with each inhibitor.(**B**) Root Mean Square Fluctuation (RMSF) analysis depicting residue-level flexibility across the EGFR variants, providing insight into local conformational dynamics upon ligand binding.(**C**) Radius of gyration (Rg) plots evaluating the compactness and structural integrity of each protein–ligand complex throughout the simulation.

(**A**)

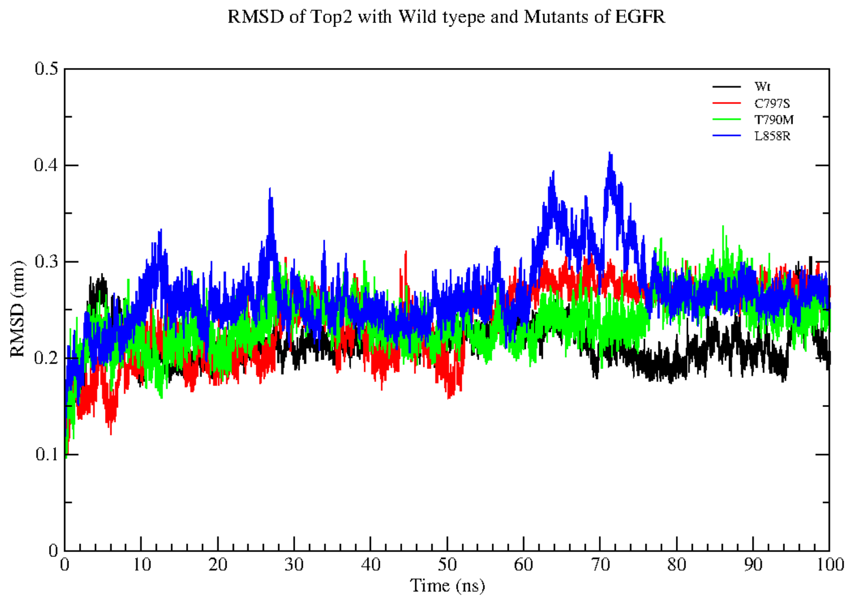
RMSD of Top2 with Wild tyepe and Mutants of EGFR
Wt
C797S
T790M
L858R
RMSD (nm)
0.5
0.4
0.3
0.2
0.1
0
0
10
20
30
40
50
60
70
80
90
100
Time (ns)

(B)

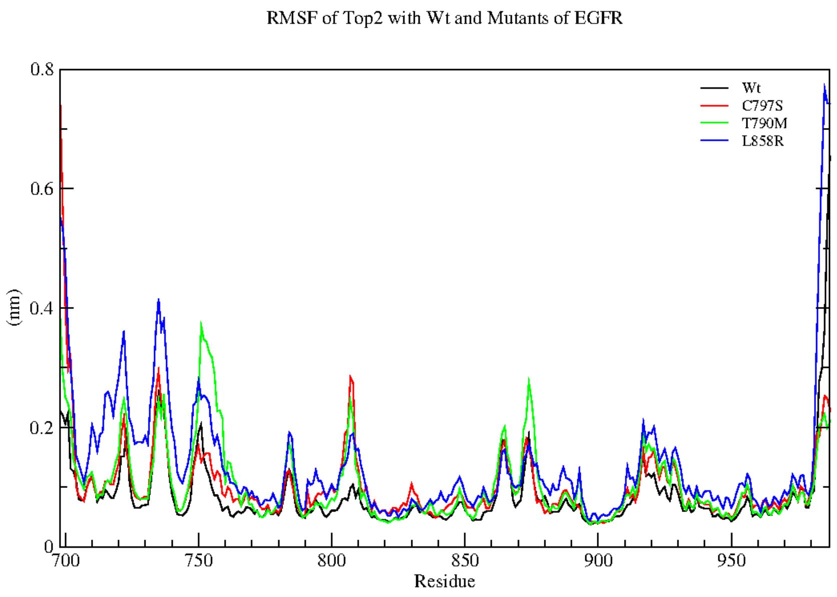
RMSF of Top2 with Wt and Mutants of EGFR
Wt
C797S
T790M
L858R
(nm)
0.8
0.6
0.4
0.2
0
700
750
800
850
900
950
Residue

(C)

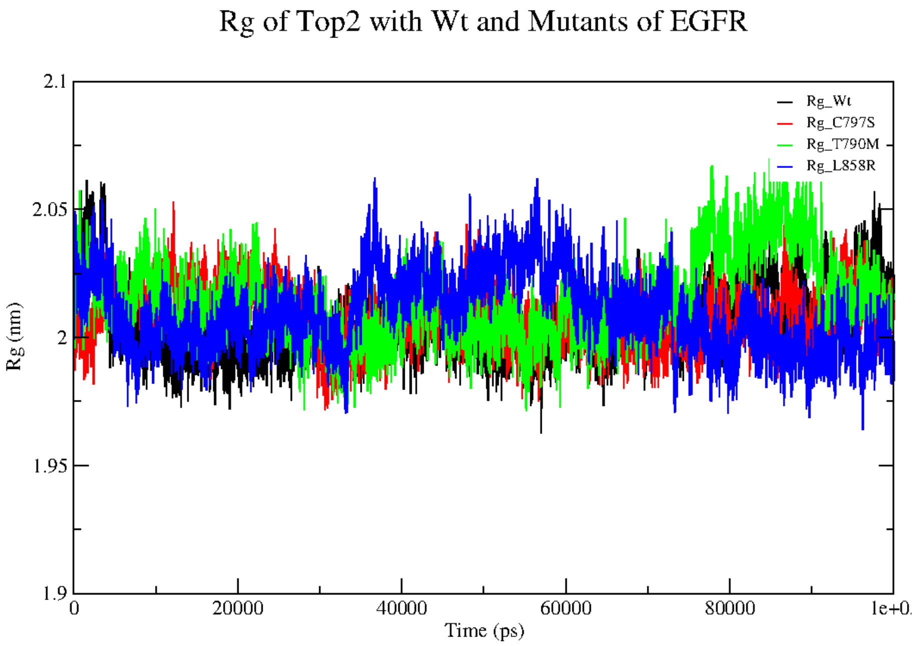


**Figure S8.** Comparative molecular dynamics analysis of EGFR wild-type and mutant complexes (C797S, T790M, L858R) with Top2 over a 100 ns simulation. (**A**) Root Mean Square Deviation (RMSD), profiles illustrating the overall structural stability and equilibration of the EGFR wild-type and mutant protein backbones in complex with each inhibitor.(**B**) Root Mean Square Fluctuation (RMSF) analysis depicting residue-level flexibility across the EGFR variants, providing insight into local conformational dynamics upon ligand binding.(**C**) Radius of gyration (Rg) plots evaluating the compactness and structural integrity of each protein–ligand complex throughout the simulation.

**Table S4: S**ummary of Binding Free Energy Calculations for mutants of EGFR with FDA approved and underinvestigated inhibitors. In this table , the calculated binding free energies and their components for two-FDA-approved two underinvestigated potential inhibitors (Afatinib, Osimertinib and Top2) using the MM-PBSA method.

| Molecule Name | Mutant_Type | ΔGtotal (kJ/mol) | ΔGele (kJ/mol) | ΔGvdw (kJ/mol) | ΔGpol (kJ/mol) | ΔGnonpol (kJ/mol) |
|---|---|---|---|---|---|---|
| Afatinib | C797S | -25.21 | -6.21 | -37.87 | 22 | -4.08 |
| | T790M | -18.79 | -6.50 | -29.87 | 21.46 | -3.89 |
| | L858R | -42.54 | -13.82 | -52.51 | 30.62 | -6.82 |
| Osimertinib | C797S | -21.68 | -5.5 | -31.02 | 33.13 | -3.97 |
| | T790M | -42.72 | -6.6 | -25.08 | 40.05 | -6.25 |
| | L858R | -25.9 | -36.20 | -36.75 | 20.78 | -4.59 |

| | | | | | | |
|---|---|---|---|---|---|---|
| Top2 | C797S | -55.94 | -50.86 | -67.69 | 72.69 | -8.09 |
| | T790M | -25.53 | -45.08 | -37.40 | 61.78 | -4.83 |
| | L858R | -45.25 | -41.73 | -42.50 | 38.30 | -4.37 |

ΔGtotal: Total binding free energy, representing the overall energy change upon ligand binding. ΔGele: Electrostatic contribution to the binding free energy, reflecting the charged interactions between the protein and the ligand. ΔGvdw: van der Waals contribution to the binding free energy, representing the attractive and repulsive forces arising from the temporary fluctuations in electron distribution. ΔGpol: Polar solvation energy, representing the energy change associated with the desolvation of polar and charged groups upon complex formation. ΔGnonpol: Non-polar solvation energy, representing the favorable energy change associated with the burial of non-polar surface area upon binding.

**Table S5:** It shows the predicted binding free energies (ΔG) obtained from molecular dynamics simulations, which were validated by comparison with experimentally reported binding affinities.

| FDA-Approved inhibitor | ΔG_predicted (kcal/mol) | ΔG_Experimentally_calculated (kcal/mol) | References |
|---|---|---|---|
| Afatinib | –35.2 | –33.0 | Li et al., 2008 |
| Erlotinib | –32.5 | –30.6 | Zhang et al., 2010 |
| Osimertinib | –38.7 | –36.1 | Cross et al., 2014 |
